# Antibacterial and anticancer properties of *Solanum mauritianum* fruit components analyzed using LC-QTOF-MS/MS

**DOI:** 10.1038/s41598-025-01348-w

**Published:** 2025-05-14

**Authors:** Abraham Goodness Ogofure, Tendani Sebola, Ezekiel Green

**Affiliations:** Molecular Pathogenic and Molecular Epidemiology Research Group (MPMERG), Department of Biotechnology and Food-Technology, Faculty of Science, University of Johannesburg, P. O. Box 17011, Doornfontein, Johannesburg, 2028 South Africa

**Keywords:** Cytotoxicity, Antimicrobial resistance, Anticancer, Secondary metabolites, Chromatography, Biotechnology, Cancer, Drug discovery, Microbiology, Plant sciences, Diseases, Chemistry

## Abstract

This study evaluated the antibacterial and anticancer properties of *S. mauritianum* fruit components through LC-QTOF-MS/MS metabolomic profiling. The samples were extracted, and the antibacterial activity was conducted using a standard Resazurin microtiter assay. The minimum inhibitory concentrations (MICs) of the crude extracts were evaluated against reference pathogenic bacterial isolates. The anticancer activity of the extracts was tested against U-87 MG glioblastoma and A549 lung carcinoma cells (ATCC cancer cell lines). The real-time toxicity assay and comprehensive metabolomic profiling were evaluated for the crude extracts. Results revealed that the ripe fruit coat exhibited the richest chemical diversity, with 15 unique metabolites, while the unripe fruit had 5. Detailed classification of the identified metabolites showed that alkaloids accounted for 33.3%, followed by terpenoids (21.2%). The extracts of the fruit components had significant antibacterial activity against the referenced pathogens of public health importance. Extracts from the ripe fruit coat demonstrated significant cytotoxicity on U-87 MG glioblastoma cell viability, suggesting potential anticancer activity, while the effect on A549 lung carcinoma cells showed high viability across all treatments. The real-time cytotoxicity assays further highlighted the dose-dependent inhibition of glioblastoma cells by crude extracts from the ripe fruit coat, emphasizing its therapeutic potential.

**Supplementary Information:**

The online version contains supplementary material available at 10.1038/s41598-025-01348-w.

## Introduction

Globally, the burden of antibiotic-resistant bacterial infections and cancer continues to escalate, posing significant challenges to public health and the general healthcare systems^1–4^. As conventional therapeutic approaches face mounting limitations, there is an urgent need to explore novel, naturally derived compounds with the potential for antibacterial and anticancer properties. In this context, plant-based secondary metabolites have emerged as a promising frontier in drug discovery, offering a vast and largely untapped reservoir of bioactive compounds^5–9^.

*Solanum mauritianum* Scop., an invasive species commonly known as woolly nightshade or bugweed, is a plant species that has garnered increasing attention in ethnopharmacological research^10–12^. While traditionally used in folk medicine across various cultures^10,13^. Several critical studies have evaluated the woolly nightshade’s pharmacological and medicinal properties. It was one of the medicinal plants employed for treating and managing cancer in Kenya^14^. In some parts of West Africa, the plant is used to treat ailments like yaws, ulcers, hemorrhoids, and wound infection^15^. Diverse bioactive compounds reported from *S. mauritianum* contribute to its potential medicinal applications, including steroidal alkaloids, flavonoids, phenolics, tannins, and saponins^13^. The plant has been reported to exhibit significant antimicrobial activity against Gram-positive and Gram-negative bacteria and certain fungi through its bioactive compounds and endophytes^7,16–18^. The full therapeutic potential of *S. mauritianum*, particularly its fruit coat, remains largely unexplored in modern analytical techniques. This oversight represents a significant gap in our understanding, especially considering the plant’s widespread distribution and the potential for sustainable harvesting of its fruit coat.

The fruit coat of *S. mauritianum* is of particular interest due to its rich phytochemical profile, which is hypothesized to contain a diverse array of bioactive compounds. These may include alkaloids, phenolic compounds, and other secondary metabolites known for their potential antimicrobial and antiproliferative activities^13,19^. However, a comprehensive characterization of these compounds and their biological activities has yet to be conducted using state-of-the-art analytical methods.

Liquid chromatography coupled with quadrupole time-of-flight mass spectrometry (LC-QTOF-MS/MS) represents a cutting-edge approach to metabolomic profiling, offering unparalleled sensitivity and resolution in identifying and quantifying complex chemical mixtures^20–22^. By employing this advanced technique, we aim to provide a detailed map of the phytochemical landscape present in the *S. mauritianum* fruit coat, potentially uncovering novel compounds or unique combinations of metabolites responsible for its putative therapeutic effects. The integration of untargeted metabolomic profiling of promising compounds with targeted bioassays for antibacterial and anticancer activities offers a powerful strategy for the discovery and identification (with high sensitivity) of potential lead compounds^19,20,22^. This strategy combines the breadth of comprehensive metabolite analysis with the specificity of functional testing, enabling the identification of bioactive compounds that traditional screening methods might overlook^20^. By simultaneously mapping the chemical landscape of complex extracts from the fruit coats of *S. mauritianum* and evaluating its biological effects, we can efficiently pinpoint potential lead compounds, understand structure-activity relationships, and even uncover synergistic interactions between metabolites. This approach accelerates the discovery process and provides deeper insights (with further studies) into the mechanisms underlying the observed antibacterial and anticancer properties, paving the way for more informed and effective drug development strategies.

Furthermore, our study aligns with the growing emphasis on sustainable and eco-friendly approaches to drug discovery. By focusing on the fruit coat, a part of the plant that is often discarded, we advocate for the full utilization of natural resources and the potential development of value-added products from agricultural waste streams. This approach resonates with circular economy principles and could potentially offer new economic opportunities for communities where *S. mauritianum* is abundant.

In light of these considerations, we comprehensively investigated the antibacterial and anticancer properties of *S. mauritianum* Scop. fruit coat through LC-QTOF-MS/MS metabolomic profiling. By bridging the gap between traditional knowledge and modern analytical techniques, we sought to uncover novel bioactive compounds and evaluate their potential as leads for drug development. This research contributes to the growing body of knowledge on plant-based therapeutics and paves the way for innovative approaches in combating antibiotic resistance and cancer treatment, two of the most pressing health challenges of our time. Through this multifaceted approach, we aspire to significantly contribute to the fields of natural product chemistry, pharmacognosy, and drug discovery, potentially uncovering new avenues for therapeutic interventions and setting the stage for future translational research in the ongoing war against bacterial infections and cancer.

## Methodology

### Collection of plant samples (*Solanum mauritianum*)

The fruits and leaves of *Solanum mauritianum* were collected from the University of Johannesburg’s Doorfontein campus and formally identified by Professor Annah Moteetee, Dean of the Faculty of Science. A voucher specimen (BTNPSP02) has been deposited in the publicly accessible herbarium of the University of Johannesburg. The fruits were separated into different categories, such as ripe fruits and unripe fruits. The fruits’ seeds and the coat of ripe fruit were also separated for immediate analysis at the Molecular Pathogenic and Molecular Epidemiology Research Group (MPMERG) at the University of Johannesburg, Doornfontein Campus. The samples were washed several times with sterilized distilled water, and complete surface sterilization of the samples was achieved using absolute ethanol. The specimens were dried, wrapped in aluminum foil and kept for further analysis.

## Extraction of plant part

The dried samples were blended and weighed using a commercial blender, and two hundred grams (200 g) of the blended portion from each sample was used for extraction following the methods delineated by Fomogne-Fodjo et al.^23^ with little modifications. Into a 3000 ml capacity Schott bottle containing a 2000 mL mixture of chloroform and methanol (1:1 or v/v or 50/50 portion), the weighted portion of the sample was added and left on an IKA^®^ Rocker 3D basic (Inqaba Biotec, SA) for 48 h. The suspended extracts were filtered with the aid of Whatman No. 1 filter paper, and the resulting solvents in the mixture were evaporated at 45 ^0^C in a VP30 LabTech rotary evaporator (Hopkinton, Massachusetts). The process was repeated twice to enhance the extraction of compounds, and the resulting crude extract was collected in an amber bottle and placed in a desiccator for proper drying.

## Determination of antibacterial activity

The antibacterial activity and corresponding minimum inhibitory concentration (MIC) were evaluated in the study using a standard Resazurin Microtitre Assay according to methods delineated by Sebola et al.^24^ and Pelo et al.^25^ with slight modification. The antibacterial activity and MICs of the crude plant extracts of the ripe fruits seed, ripe fruit coat and unripe fruits were evaluated against reference bacterial isolates, which include *Escherichia coli* (ATCC 10536), *Mycobacterium smegmatis* (ATCC 21293), *Bacillus cereus* (ATCC 10876), *Streptococcus epidermidis* (ATCC 14990), *Klebsiella pneumonia* (ATCC 10031), *Bacillus subtilis* (ATCC 19659), *Mycobacterium marinum* (ATCC 927), *Staphylococcus aureus* (ATCC 25923), *Enterobacter aerogenes* (ATTC 13048), *Pseudomonas aeruginosa* (ATCC 10145), and *Proteus vulgaris* (ATCC 33420). Serial dilutions of the crude extract were prepared from 8 mg/mL to 0.25 mg/mL from the stock concentration in 0.1% dimethyl sulfoxide (DMSO). The outermost wells of plates were horizontally and vertically filled with 100 µL of 0.5 McFarland’s standard (corresponding to 1.5 × 10^8^ cells/mL) bacterial suspension grown overnight and sterile distilled water, which served as controls. The experiment was conducted in five independent biological replicates for each plant extract. Vertically, 100 µL of the diluted plant extract beginning with 8 mg/mL were pipetted into the wells until a 0.125 mg/mL concentration was reached. 100 µL of the bacterial suspension was added to these wells (1:1 v/v), bringing the concentrations of the extracts from 4 mg/mL to 0.063 mg/mL (following the addition of the bacteria to the wells containing the extract in a 1:1 ratio). Incubation of the plates was done for 18–24 h. at 37 °C before adding 10 µL of 0.02% (w/v) solution of sodium salt (resazurin) to the wells, which was further covered with aluminum foil and incubated for a further 2 h. at the same temperature. Aside from the negative control in distilled water, the antibiotic streptomycin (10 µg/mL) was used as the positive control due to its broad-spectrum antibacterial activity and well-established efficacy against Gram-positive and Gram-negative bacteria. A stock solution of streptomycin was prepared by dissolving the antibiotic in sterile distilled water, followed by serial dilutions to match the test concentrations. Negative controls consisted of wells containing bacterial suspension with 0.1% DMSO (vehicle control) to confirm that the solvent had no inhibitory effects on bacterial growth. In addition, a blank control (wells containing only broth and resazurin without bacteria or extract) was included to ensure that no spontaneous colour change occurred in the absence of metabolic activity. The presence of metabolic activity or bacterial growth in the wells was indicated by a change in the colour of resazurin from blue to pink. Therefore, wells where the concentration had a noticeable colour change after the addition of resazurin were ignored (indicative of bacteria growth or resistance to antibiotics), but wells where no visible or noticeable colour change was observed were regarded as MIC^24,25^. It is worth noting that all bacterial isolates followed basic culture and growth conditions except for the *Mycobaterium* species used in the study. They were inoculated into a separate broth (Middlebrook 7H9 broth), which was treated with a growth supplement (Middlebrook OADC), before being incubated for 14 days at 30 °C (for *M*. *marinum*) and 37 °C for 24 h. (for *M*. *smegmatis*).

## Anticancer assays

The extracts from the fruit parts and seed were tested against referenced (ATCC (Manassas, VA, USA)) cancer cell lines of the brain (U-87 MG glioblastoma cell) and the lungs (A549 lung carcinoma cell). In this assay, the methods of Ogofure and Green^7^ and Sebola et al.^24^ were followed with slight modification, where 0.1% DMSO was added to a weighed crude extract sample in the Eppendorf tube and sonicated to aid the dissolution of the extract. The referenced cancer cell lines (1 × 10^5^ cells/mL), U-87 MG glioblastoma and A549 lung carcinoma (ATCC, Manassas, VA, USA) were cultured using standard tissue culture techniques. The cells were maintained in Dulbecco’s Modified Eagle Medium (DMEM) (Merck, Johannesburg, SA), supplemented with 10–15% fetal bovine serum (FBS) (Merck, Johannesburg, SA), under standard conditions (37 °C) in a humidified atmosphere containing 5% CO₂ to mimic physiological conditions. A 200 µg/mL stock solution was prepared, which was then serially diluted (double fold) using the growth media (1:1 v/v) until a concentration of 6.25 µg/mL was obtained. This would make the final concentrations run from 100 µg/mL to 3.13 µg/mL. The in-vitro cytotoxicity assay employed involves the assessment of cell viability via a colour change from yellow (an MTS (3 (4,5-dimethylthiazol-2-yl)−5-(3-carboxymethoxy-phenyl)−2-(4-sulfophenyl)−2 H-tetrazolium) compound) to dark purple solution, which is visible at 490 nm under an Eppendorf AG 2231 Biospectrometer (Hamburg, Germany). The change in colour from yellow to dark purple would typically occur when the MTS gets metabolized by viable cells, and the cell viability of the samples shares a direct proportional relationship with the measured absorbance at 490 nm. In this assay, triplicate plates were used for each sample, which was further evaluated in duplicate to give a biological replicate of 6 per sample. The cells were incubated for 18–24 h. at 37 °C before the addition of the supplied compounds in varying concentrations from 100 µg/mL to 0 µg/mL and then left to further incubate for 4 days before adding 5 µL of MTS to the cells and measuring the absorbance at 490 nm using a UV-VIS spectrophotometer (Eppendorf AG 2231 Biospectrometer) after a further varying period of incubation (1, 2, and 4 h. respectively). The positive control employed in the assay was auranofin, which, according to Li et al.^26^ and Roder and Thomson^27^, was reported to have excellent cytotoxic activities against non-small cancer cell lines. The formula employed for evaluating the viability of the cells is shown below:

$${\%}\:\text{Cell}\:\text{Viability}=\frac{\text{E}_\text{a}-\text{B}_\text{a}}{\text{C}_\text{a}-\text{B}_\text{a}}\times 100$$

According to Handayani et al.^28^, *E*_*a*_ stands for the absorbance of the extract, *B*_*a*_ is the absorbance of the blank, and *C*_*a*_ is the absorbance of the control.

## Real-time cytotoxicity assay (RTCA) of U-87 MG cells using xCELLigence^®^ real-time cell analyzer

The xCELLigence^®^ RTCA assay involved seeding the glioblastoma cell line (U-87 MG) at a standard density of 1 × 10^5^ cells/mL into 96-well electronic plates pre-coated with a gold microelectrode (E-Plate^®^ 96; ACEA Biosciences Inc., San Diego, CA, USA) before incubation in 5% CO_2_ at 37 °C as delineated by Man et al.^29^. Following the aforementioned, *S. mauritianum* fruit coat crude extract of the fruits coat was dissolved in 0.1% DMSO and then added at concentrations of (100 µg/mL, 50.0 µg/mL, 25.0 µg/mL, 12.5 µg/mL, 6.25 µg/mL, and 3.125 µg/mL) in triplicates. The cells were further incubated for 96 h, with impedance measurements recorded every 15 min. A graphical representation of the resulting toxicity data was generated for analysis.

### Untargeted metabolomic profiling of the extracts by LC-QTOF-MS/MS

The untargeted metabolomic profiling of the crude extracts from the fruits of *S. mauritianum* was evaluated following methods described by Tapfuma et al.^30^ and Tapfuma et al.^31^. The crude extract was dissolved in methanol (HPLC grade, Merc, South Africa) at a concentration of 1 mg/mL, and the crude extract-methanol mixture was sonicated until full dissolution (for about 8–10 min.) was achieved. Following complete dissolution, the samples were filtered using a PVDF (polyvinylidene fluoride) syringe of 0.22 μm size into 1 mL capacity LC-MS autosampler vials. The blank used for proper calibration of the system was the HPLC-grade methanol devoid of the analyte. These blanks were run under the same conditions as the sample extracts to account for any contributions from the filter material, and the results were taken into consideration when analyzing the data. The comprehensive analysis of the crude secondary metabolites was conducted using an ultra-high-performance LC-QTOF-MS system. The LC system used in our study was a Dionex UltiMate 3000 UHPLC (Thermo Scientific, Darmstadt, Germany), which was coupled to a Compact™ QTOF (Bruker Daltonics, Bremen, Germany) Mass Spectrometer. The system uses electrospray ionization (ESI) for the analysis, and the analytes were separated chromatographically using reverse phase ultra-high-performance liquid chromatography (RP-UHPLC) with a 5µL injection volume. A Raptor ARC-18 column (Restek, USA) was used for chromatographic separation, and this ARC-18 column had a length of 100 mm, an internal diameter of 2.1 mm, a particle size of 2.7 μm, and a pore size of 90 Å. The HyStar software version 2.10 (Bruker, Germany) controlled the system, collected data, and managed the entire analysis process. The mobile phases were 0.1% formic acid in acetonitrile (solvent B) and 0.1% formic acid in water (solvent A). The entire chromatographic run lasted for 40 min., with the gradient beginning at 5% solvent B for 2 min., before increasing to 95% solvent B for over 28 min., remaining isocratic at 95% solvent B for 5 min., and then decreasing to 5% solvent B in 1 min.

## LC-QTOF-MS settings

Metabolomic profiling was conducted using a Bruker QTOF Series 1.9 LC-QTOF-MS Compass system (Impact II, Bruker Daltonics GmbH, Baden-Württemberg, Germany) for identifying secondary metabolites in the crude extracts of *S. mauritianum*. Chromatographic separation was performed on a RESTEC model reverse-phase C18 column (50 mm × 2.1 mm, 1.7 μm particle size) with a mobile phase which consists of 0.1% formic acid in acetonitrile and water. Mass spectrometry data were acquired using electrospray ionization (ESI) in positive mode, scanning between 50 and 1600 m/z. The instrument was set to a nebulizer pressure of 1.8 bar, a dry gas flow rate of 8 L/min, and a capillary voltage of 4500 V. The column flow rate was maintained at 0.3 mL/min, with a draw speed of 2 µL/s and an injection volume of 2 µL. The column oven temperature was set at 35 °C. The mass spectrometer (MS) was configured with the following parameters: gas temperature of 220 °C, drying gas flow of 8 L/min, ionization energy of 4.0 eV, collision energy of 7.0 eV and a cycle time of 0.5 s. High-resolution LC-QTOF-MS/MS data were acquired in centroid mode with internal lock mass calibration to ensure accurate m/z values and minimal drift. Feature detection and mass peak extraction were conducted using Bruker Compass DataAnalysis software (version 4.3). Mass peak detection was performed with a mass accuracy tolerance of ± 5 ppm for precursor and fragment ions. Retention time alignment across all samples was carried out using a tolerance window of ± 0.2 min, ensuring reliable comparison of metabolite elution profiles between sample groups.

Data processing was conducted using Bruker Compass Data Analysis 4.3, and the identification of the compounds was performed by cross-referencing spectral data with the National Institute of Standards and Technology (NIST), 2005 library and major metabolomic databases, including PubChem, KEGG, MetFrag, ChemSpider, and ChEBI. Peak identification was conducted based on accurate mass measurements, MS/MS fragmentation patterns, and retention time (r.t.). The acquired spectral data were cross-referenced with multiple databases, including Kyoto Encyclopedia of Genes and Genomes (KEGG), PubChem, ChemSpider, MetFrag, and Chemical Entities of Biological Interest (ChEBI), to ensure accurate metabolite annotation. Peak deconvolution was carried out within the Bruker Compass environment using its recursive feature extraction module. The following parameters were applied: a signal-to-noise ratio threshold of 3:1, a minimum peak width of 5 scans, and Savitzky-Golay smoothing (polynomial order 2, window size of 7 points). Baseline correction was performed using the rolling ball algorithm with a 10% noise band to enhance peak purity and minimize false positives resulting from background noise or overlapping signals. Putative annotation of secondary metabolites was achieved by matching precursor ions at specific *m/z* values with corresponding MS/MS fragment ions, ensuring high confidence in compound annotation^18^.

## Data analysis and visualization

Our research utilized the Bruker Compass software (version 4.3) to interpret spectral data, and MetFrag (version 2.1) was employed for fragment spectrum characterization linked to public databases such as PubChem, ChemSpider, and KEGG. All compounds reported in this study were annotated based on spectral library matches and were not confirmed with analytical standards. Therefore, annotations reflect Level 2 confidence based on the Metabolomics Standards Initiative (MSI) guidelines^32^ as aforementioned.

Compounds with mass accuracy within ± 5 ppm, MS/MS spectral similarity with a cosine score ≥ 0.7 when compared to reference spectra, and a retention time within ± 0.2 min of database or literature-reported values were retained for annotation. Customized settings were applied for the LC-QTOF-MS for more accurate results. Given the nature of the data and research objectives, descriptive statistics and visual representations were employed to explore, summarize and interpret the data obtained in this study. The annotation of metabolite was conducted according to the Metabolomics Standards Initiative (MSI) guidelines as described by Sumner et al.^32^. All the annotated compounds in our study are classified as Level 2 confidence based on accurate mass, MS/MS fragmentation patterns and retention time comparison public databases (aforementioned).

The antibacterial and cytotoxic activity data were visualized using R studio (version 4.3.3) following Ogofure et al.^18^ and Ogofure and Green^7^ visualization pattern with specialized R packages^33^. The *heatmap* package^34^ facilitated the generation of heatmaps to display metabolite profiles and cytotoxicity results without incorporating hierarchical clustering and custom colour scales. Enhanced visualizations, including heatmaps with numerical data, were created using the *ggplot2* package^35^ and *cowplot*, allowing for detailed control over aesthetics. For the comparison of the secondary metabolites among plant parts, a Venn diagram was constructed using the *VennDiagram* package^36^, featuring custom label positions and colour schemes for clarity. Statistical analysis, including clustering and correlations, was performed using base R functions and additional packages as needed. Visualizations were carefully optimized for clarity, with attention to colour, font size, and layout for effective presentation of the data^7^. The data were reshaped into a long format using tidyverse, and a two-way ANOVA was performed to evaluate the effects of plant parts and extract concentrations on cell viability, including their interaction. Assumptions of normality and homogeneity of variances were verified using the Shapiro-Wilk and Levene’s tests, respectively. To determine pairwise differences among factor levels, post hoc comparisons were conducted using Tukey’s Honest Significant Difference (HSD) test. All results were considered significant at p *< 0.05*. Descriptive and inferential visualizations were produced using the ggpubr and ggplot2 packages.

## Results and discussion

**Fig. 1:**
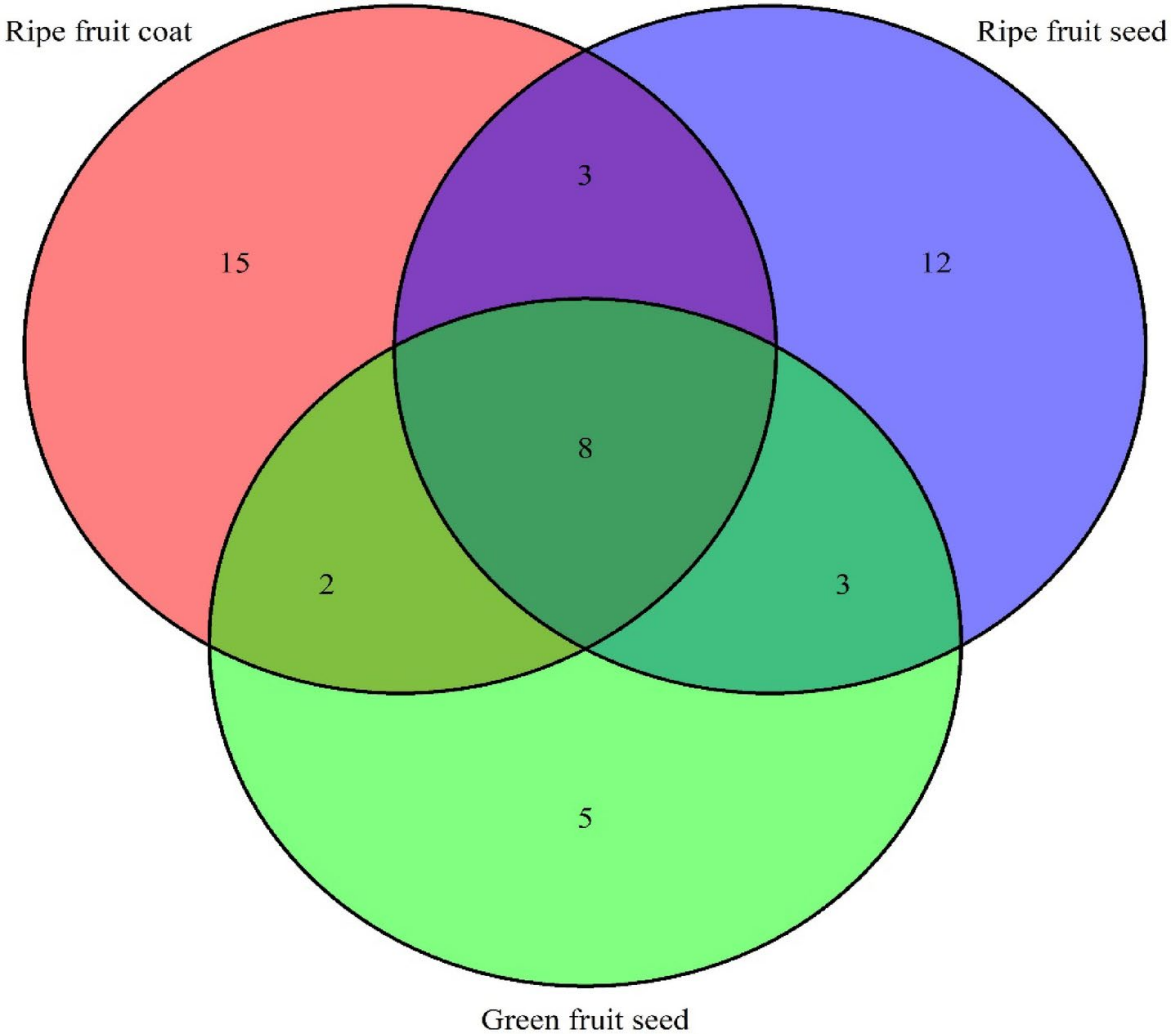
Distribution of secondary metabolites across the fruits of *S. mauritianum* plants.

The Venn diagram (Fig. 1) presented in this study provides a detailed visualization of the distribution of secondary metabolites across the fruits (ripe and unripe) of the *S. mauritianum* plant, specifically the ripe fruit coat, ripe fruit seed, and green fruit seed. The ripe fruit coat exhibits the highest unique metabolite content, with exclusively 15 distinct secondary metabolites associated with the ripe fruit coat, indicating a rich chemical diversity that could contribute significantly to its bioactive properties. The ripe fruit seed contains 12 unique metabolites, while the green fruit seed (unripe) has the lowest unique metabolite count with 5 metabolites. Notably, the overlap between the ripe fruit coat and the ripe fruit seed reveals 3 shared metabolites, suggesting a degree of biochemical similarity between these two parts. Furthermore, an intersection of all three plant parts shows 8 common secondary metabolites extracted using a 50:50 methanol and chloroform combination, which may represent core compounds essential to the plant’s overall metabolic framework and adaptive responses. It is important to note, however, that using other solvent combinations might have resulted in the extraction of additional metabolites, offering a broader profile of the plant’s chemical composition. A 50:50 methanol and chloroform combination was selected for further analysis due to its demonstrated antibacterial activity, making it the most effective option for this study. However, this solvent system has inherent limitations, as it may favour the extraction of mid-polar and nonpolar metabolites while potentially excluding highly polar compounds. More so, the use of chloroform, while highly effective in our study for extracting lipophilic compounds, presents significant concerns due to its environmental toxicity and potential health hazards, necessitating cautious handling and consideration of greener alternatives. Future research, therefore, should explore alternative solvent combinations, such as ethyl acetate-methanol-water or n-hexane-ethyl acetate-methanol, to facilitate a broader metabolite profile encompassing a wider range of polarities.

Additionally, advanced extraction techniques, such as supercritical fluid extraction (SFE) or deep eutectic solvents (DES), may provide a more environmentally friendly and efficient approach for obtaining diverse secondary metabolites. The unique and shared metabolite profiles revealed by the Venn diagram provide crucial insights into the phytochemical composition of *S. mauritianum*, underscoring the potential of these plant parts for yielding bioactive compounds of pharmaceutical and medical interest. The visual clarity and the distinct separation of metabolite presence in this diagram make it an invaluable tool for guiding targeted extraction and further exploration of the medicinal properties inherent in each specific plant part.

**Fig. 2:**
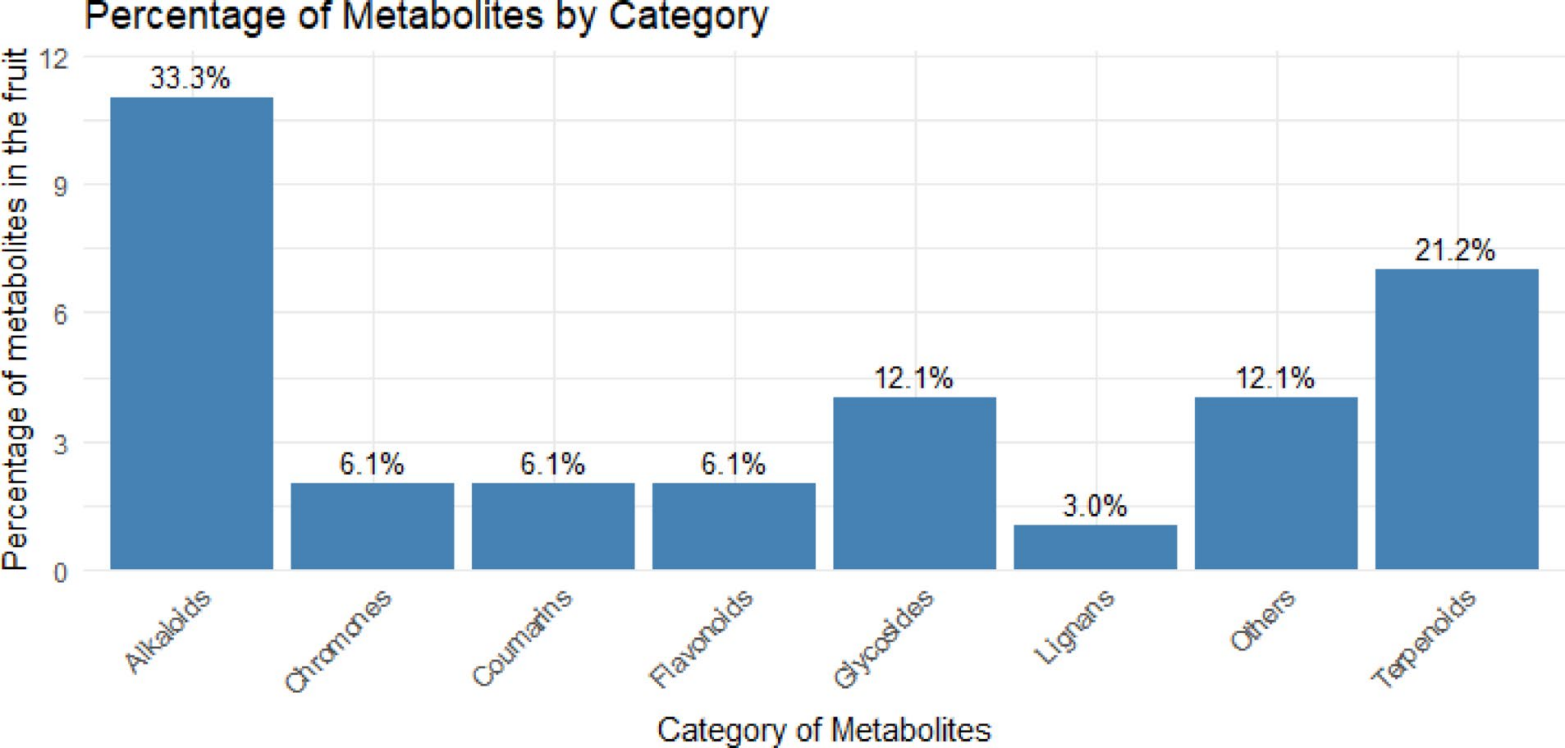
Categories of secondary metabolites across the fruits of *S. mauritianum* plants.

A comprehensive categorization by classes of the secondary metabolites identified in the fruits of *S. mauritianum* is shown in Fig. 2. The results revealed that alkaloids constitute the largest group of bioactive molecules, accounting for 33.3% of the total metabolites, highlighting their predominant role in the phytochemical profile of the fruit. Terpenoids followed as the second most abundant category, representing 21.2% of the metabolites, underscoring their significant presence and potential contribution to the plant’s bioactivity. Glycosides and other miscellaneous metabolites each make up 12.1% of the total, indicating a diverse chemical makeup within the fruit. Notably, categories such as chromones, coumarins, and flavonoids each contribute 6.1% to the metabolite profile, while lignans are the least represented, comprising only 3.0%. This distribution not only emphasizes the chemical diversity inherent in *S. mauritianum* fruits but also points to the potential pharmacological importance of these specific metabolite groups. The prominence of alkaloids and terpenoids, in particular, may be indicative of their roles in the plant’s defenses mechanisms and therapeutic potential, warranting further investigation into their bioactive properties.

Although this study is one of the pioneer studies concerning the metabolomics and production of secondary metabolites in the fruit parts of *S. mauritianum*, the findings in this study were, however, found to be consistent with the report of Maynard et al.^37^, who mentioned that ripe and unripe fruit pulp of *Piper sanctifelicis* had a plethora of specialized metabolites of varying concentrations and diversity. They further asserted that the production of these secondary metabolites is one of the plant’s most remarkable characteristics. The few studies on *S. mauritianum* by Uche-Okereafor et al.^38^, Pelo et al^25^. and^19^ concentrated on endophytes obtained from different plant parts, and the findings revealed the presence of specialized metabolites by these endophytes. There is a strong link between the production of secondary metabolites by endophytes and the production of secondary metabolites by different plant parts. The endophytes can interact with their host plants in various ways, influencing the production and composition of secondary metabolites^18^. Similarly, the report of Jayakumar and Murugan^13^ also corroborated our findings in this study as they reported that the plant *S. mauritianum* contains a substantial amount of specialized/secondary metabolites such as tannins, phenols, saponins and alkaloids, indicating its potential for external therapeutic use and other medicinal applications. More so, panoramic views on the secondary metabolites produced by *Ziziphus mauritiana* revealed that it also contains a plethora of active secondary metabolites with potential pharmacological and medicinal properties^39,40^. Additionally, Br-Pa et al.^17^ also revealed that the aerial parts of *S. mauritianum* contain pharmacologically active components which were identified to be alkaloids, saponins and flavonoids, similar to the classes of compounds identified in the fruit part of *S. mauritianum.* A detailed annotation of secondary metabolites across the fruits of *S. mauritianum* Scop. using LC-QTOF-MS/MS metabolomic profiling are shown in Table 1. The result revealed a distribution of 48 distinct metabolites across three parts of the fruit, with each metabolite characterized by its retention time (Rt), error in parts per million (Err), molecular weight ([M + H] + *m/z*), and its presence (+) or absence (−) in the respective fruit parts. The ripe fruit coat exhibited the highest diversity, containing the most metabolites, including significant compounds such as cardiospermine, cusparine, and solasonine.

**Table 1:** Annotated secondary metabolites across the studied fruits of *S. mauritianum*.

S/*N*	Metabolite name	Rt (min)	[M + H]+ (m/z)	Err (ppm)	Ripe Fruit Coat	Ripe Fruit Seed	Green Fruit Seed
1	Cardiospermin	1.27	276	−12.6	**+**	**−**	**−**
2	Furofoline I	1.36	266	−10.1	**+**	**−**	**−**
3	Riccionidin A	1.55	286	−9.4	**+**	**−**	**−**
4	Bergenin	2.13	329	2.7	**+**	**−**	**−**
5	Cusparine	3.21	308	−8.8	**+**	**−**	**−**
6	Loganin	3.17	391	−6.9	**+**	**−**	**+**
7	Theogallin	5.14	345	−7.8	**+**	**−**	**−**
8	Homoeriodictyol chalcone	5.14	303	−8.9	**+**	**−**	**−**
9	Cimifugin	5.88	307	6.8	**−**	**−**	**+**
10	Berberastine	6.21	353	−7.6	**+**	**−**	**−**
11	Scopoletin	6.61	193	−4.7	**+**	**+**	**+**
12	Lysergic acid	7.09	269	−3.3	**+**	**−**	**−**
13	Glycophymoline	7.28	251	6.8	**+**	**−**	**−**
14	Danielone	7.54	213	−12.8	**−**	**+**	**−**
15	Solasonine	8.39	884	−3.1	**+**	**+**	**+**
16	α-Solanine	8.64	868	−3.1	**+**	**+**	**+**
17	Imperialine	8.69	430	−6.3	**+**	**+**	**+**
18	Genipin	9.06	227	8.8	**+**	**+**	**−**
19	Solasodine	9.83	414	−6.5	**+**	**+**	**+**
20	α-Ergocryptine	10.11	576	−4.7	**+**	**+**	**+**
21	Dioscin	10.11	869	−3.1	**−**	**−**	**+**
22	Podolide	11.12	331	−8.2	**−**	**+**	**−**
23	Esculetin	11.24	179	−5	**−**	**+**	**−**
24	Callicarpone	12.57	333	5.1	**+**	**−**	**−**
25	10-Deoxysarpagine	12.6	295	−9.1	**+**	**+**	**+**
26	Montanol	16.64	353	−7.6	**+**	**+**	**+**
27	Glycobismine A	17.26	603	−0.8	**−**	**+**	**−**
28	Coniferyl alcohol	18.37	181	−4.4	**−**	**+**	**−**
29	Hypercalin B	18.88	519	−6.2	**+**	**−**	**−**
30	Ribalinium	18.92	291	−7.9	**−**	**+**	**−**
31	Rhododendrin	19.04	329	−4.6	**−**	**+**	**−**
32	Eugenin	19.46	207	−13	**+**	**+**	**−**
33	Thalicarpine	19.47	697	−3.9	**+**	**−**	**−**
34	Hetisine	20.44	330	−8.2	**−**	**+**	**−**
35	Anatabine	20.92	161	−16.8	**+**	**+**	**−**
36	Tuliposide A	21.67	279	5.4	**−**	**+**	**+**
37	Vasconine	22.67	267	3.7	**−**	**+**	**−**
38	Columbin	22.81	359	8.4	**−**	**+**	**−**
39	Lycorine	24	288	−0.3	**−**	**+**	**−**
40	Lycocernuine	24.42	279	−9.7	**−**	**−**	**+**
41	Cannabielsoin	26	331	1.5	**−**	**+**	**+**
42	Ibogamine	26.28	281	−14.6	**+**	**−**	**+**
43	Ibogaine	26.38	311	−8.7	**+**	**−**	**−**
44	Cucurbitacin E	27.01	557	−4.8	**−**	**−**	**+**
45	Absinthin	27.97	497	−11.7	**−**	**+**	**+**
46	Clivoline	28.31	406	−0.5	**−**	**+**	**−**
47	Neoquassin	30.62	391	−6.9	**−**	**−**	**+**
48	Tingenone	31.85	421	−6.4	**+**	**−**	**−**

*Key: see supplementary materials for peaks and further information.

In contrast, the ripe fruit seed and green fruit seed showed a more selective profile, with some metabolites being unique to specific parts, like solasodine in the ripe fruit seed and glycosimine A in the green fruit seed. Notably, metabolites like α-solanine, glycochymoline, and coniferyl alcohol were detected across all three fruit parts, suggesting their ubiquitous presence and potential importance in the plant’s overall biochemical profile. The data also reveal that certain metabolites, such as eugenin and tingenone, are exclusively present in the ripe fruit coat (see supplementary material), which may indicate specialized roles in the plant’s physiology or defence mechanisms. This metabolomic profiling underscores the chemical complexity of *S. mauritianum* fruits and offers valuable insights into the distribution and potential biological roles of these secondary metabolites. The findings pave the way for further exploration into the pharmacological applications of these compounds, particularly in antibacterial and anticancer studies, where specific metabolites may exhibit potent bioactivities.

The findings in this study correspond to reports by Hadacek^41^ and Farag et al.^42^, who used similar methods (LC-QTOF-MS/MS) to evaluate untargeted specialized metabolites of different plant parts and revealed a plethora of active compounds with pharmacological activities. Similarly, Pelo et al.^19^ reported the presence of similar specialized metabolites from isolated endophytes in *S. mauritianum* plant parts. They show that using a gas chromatographic technique, a plethora of volatile compounds (promising bioactive secondary metabolites) can be identified from crude extracts. Similarly, the stems of *S. mauritianum* have been reported to harbour specialized metabolites with pharmacological and medicinal properties^38^. At variance with the result obtained in this study were reports which asserted that over 100 specialized metabolites were identified in *Solanum* species using a similar chromatographic technique with about 32 new compounds with pharmacological activities^43–45^. However, the unique sets of specialized metabolites produced by plants are a distinguishable factor that determines the types of plant species and varieties^43^. Several specific compounds highlighted in this study have also been detected in other Solanum species found in different regions of the world. Solasonine and cardiospermine have been identified from *Solanum* species using high-performance chromatography, and these compounds were reported to have pharmacological activities^46–48^. One potential reason certain compounds were present in the ripe fruit coat but absent in the seeds could be linked to the distinct roles each plant tissue plays physiologically. The fruit coat serves the purpose of protection (acts as a protective barrier) against external forces such as pathogens, which may explain the localization of specific secondary metabolites involved in defence mechanisms.

Additionally, the influence of potential metabolic shifts during the fruit ripening process, including enzymatic modifications and upregulation of potential biosynthetic pathways, could contribute to the increased diversity of secondary metabolites as the fruit coat may contain compounds that aid in fruit ripening, coloration, or attracting pollinators and seed dispersers, which might not be necessary for the seeds and, therefore, the presence of several specialized metabolites. Furthermore, different metabolic pathways could be active in the fruit coat as the fruit matures, leading to a wider variety of metabolites. At the same time, seeds may primarily store stable compounds necessary for germination. It is worth noting that green fruit coats were not evaluated, which could be a limitation of this research.

**Fig. 3:**
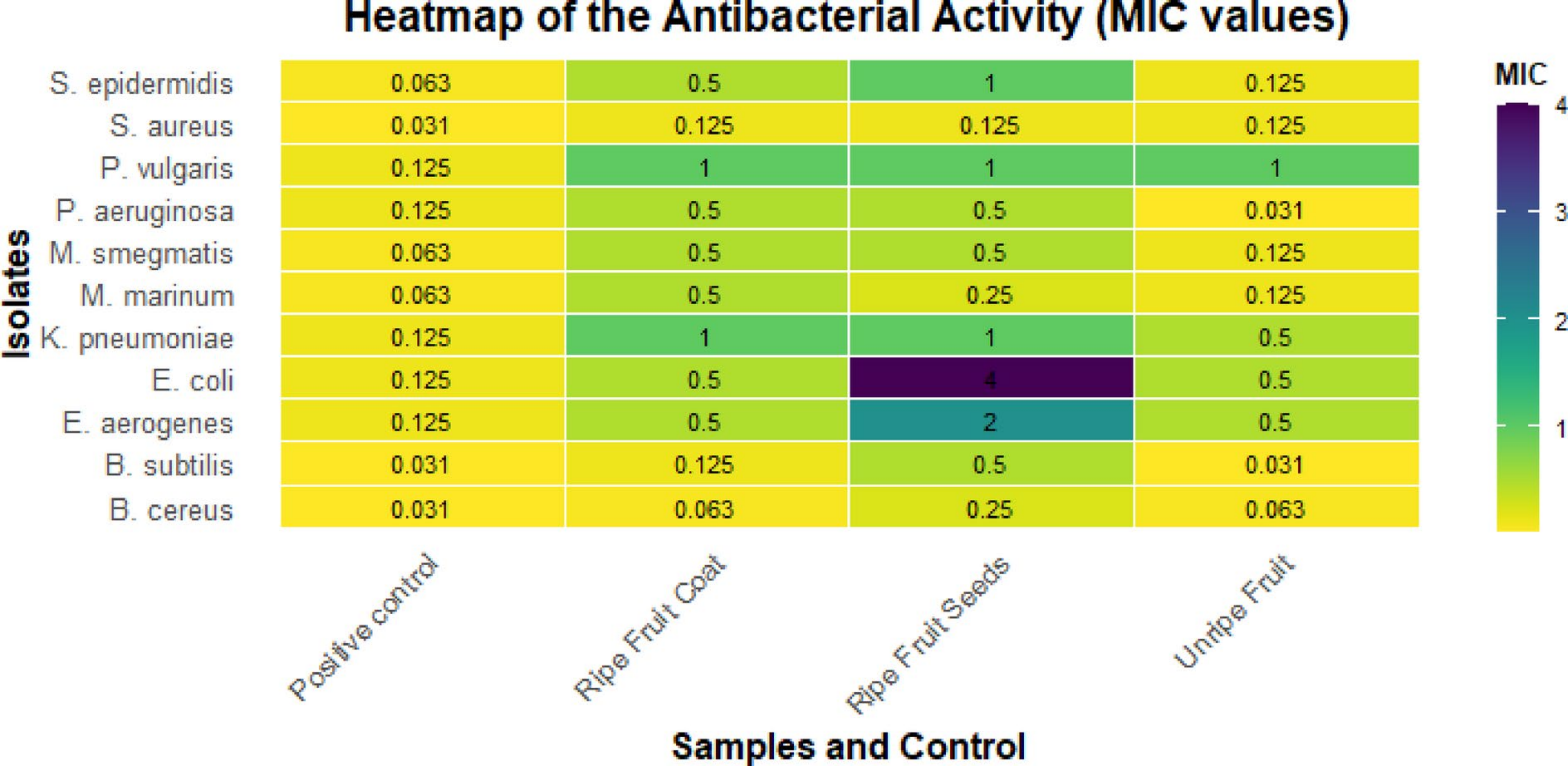
Heatmap of the antibacterial activity (mg/mL) of secondary metabolites from fruits of *S. mauritianum* Fruit components against bacterial isolates of public health importance. The positive control was streptomycin (µg/mL).

The heatmap presented in Fig. 3 shows the antibacterial activity of *S. mauritianum* fruit components. The minimum inhibitory concentration (MIC) values are displayed within each heatmap cell, reflecting the concentration at which bacterial growth was inhibited. The pathogens tested include *Bacillus cereus*, *Bacillus subtilis*, *Escherichia coli*, *Klebsiella pneumoniae*, *Mycobacterium smegmatis*, *Mycobacterium marinum*, *Proteus vulgaris*, *Pseudomonas aeruginosa*, *Staphylococcus aureus*, and *Staphylococcus epidermidis*. The tested pathogenic species are recognized for their involvement in foodborne diseases, nosocomial infections, and other areas of public health risk. The gradient scale of the heatmap emphasizes the varying degrees of antibacterial efficacy across the tested components of *S. mauritianum*, with yellow hues indicating lower MIC values and, thus, higher antibacterial activity. Notably, the fruit coat and seeds exhibited stronger inhibitory effects against Gram-positive pathogens such as *B. cereus* and *S. epidermidis*, thus suggesting that a higher concentration of bioactive compounds targeting cell wall structures possibly disrupt peptidoglycan synthesis. Whereas, the unripe fruit demonstrated moderate activity, particularly against Gram-negative bacteria like *E. coli* and *K. pneumoniae* suggests differential permeability barriers, which could influence metabolite efficacy. These findings underscore the potential of *S. mauritianum* fruit components as alternative or complementary antimicrobial agents, particularly in addressing the rising concern of antimicrobial resistance. The heatmap effectively visualizes these MIC differences, providing a comprehensive view of the plant’s antibacterial potential against a diverse array of bacterial species.

The antibacterial activity of the fruit components of *S. mauritianum* plants observed in this study was similar to the reports by Uche-Okereafor et al.^38^, who opined that the crude extracts from stems of the same plant had comparable antibacterial activity against pathogens of public health importance, such as *E. coli*,* Klebsiella pneumoniae*,* Bacillus cereus* and *Mycobacterium* species, to mention a few. Similarly, Rangasamy et al.^49^ reported that the fruit components of *S. mauritianum* showed antibacterial properties against various bacterial isolates of both Gram-positive and negative clades, with MIC values ranging between 125 and 1000 µg/mL. However, the MIC obtained in the aforementioned study was way higher than the MIC values observed in this study. The variation in MIC values could be attributed to the methods applied, environmental variations and the physiological or ontogenic state of the plant used for the experimental assay. More so, Br-Pa et al.^17^ opined that crude extracts of leaves of *S. mauritianum* were found to have an antibacterial effect against a plethora of bacterial pathogens. Aside from the limited studies in the literature concerning the antibacterial effect of *S. mauritianum* plant parts, especially the fruit components, reports of the antibacterial effect of other Solanum species are also available. These findings shed more light on the antibacterial activities of *Solanum* species with varying effects on different species of bacteria of public health importance^9,50,51^.

There are also reports concerning the antibacterial activities of crude extracts from endophytes isolated from different parts of *S. mauritianum.* It has been hypothesized that the presence of these endophytes in plant parts could play a role in the biosynthesis of these specialized metabolites in plants^7,18^. Thus, several reports revealed that crude extracts of *S. mauritianum* have been found to have antibacterial activity against bacterial species of public health importance with a broad spectrum of activity^18,25,38^. The findings of this study highlight the potential of *S. mauritianum* fruit components as valuable additions to the arsenal of antimicrobial agents, especially in the face of increasing antimicrobial resistance. This further confirms the report by Chandra et al.^52^, who opined that “plant-based antimicrobials offer a promising alternative or complementary approach to combat various pathogens without the associated side effects of synthetic drugs.” It is worthy of note that following recommendations of the Clinical and Laboratory Standard Institute, as interpreted by Ogofure et al.^18^, the MIC values for crude extracts which are less than 0.07 mg/mL are primarily considered promising for further investigation owing to the breakpoint concentrations for most antibiotics^53^. With that in mind, the fruit component of *S. mauritianum* might be very promising in search of the next antibacterial compounds that would give man the edge over multidrug-resistant bacterial isolates of public health significance.

**Fig. 4:**
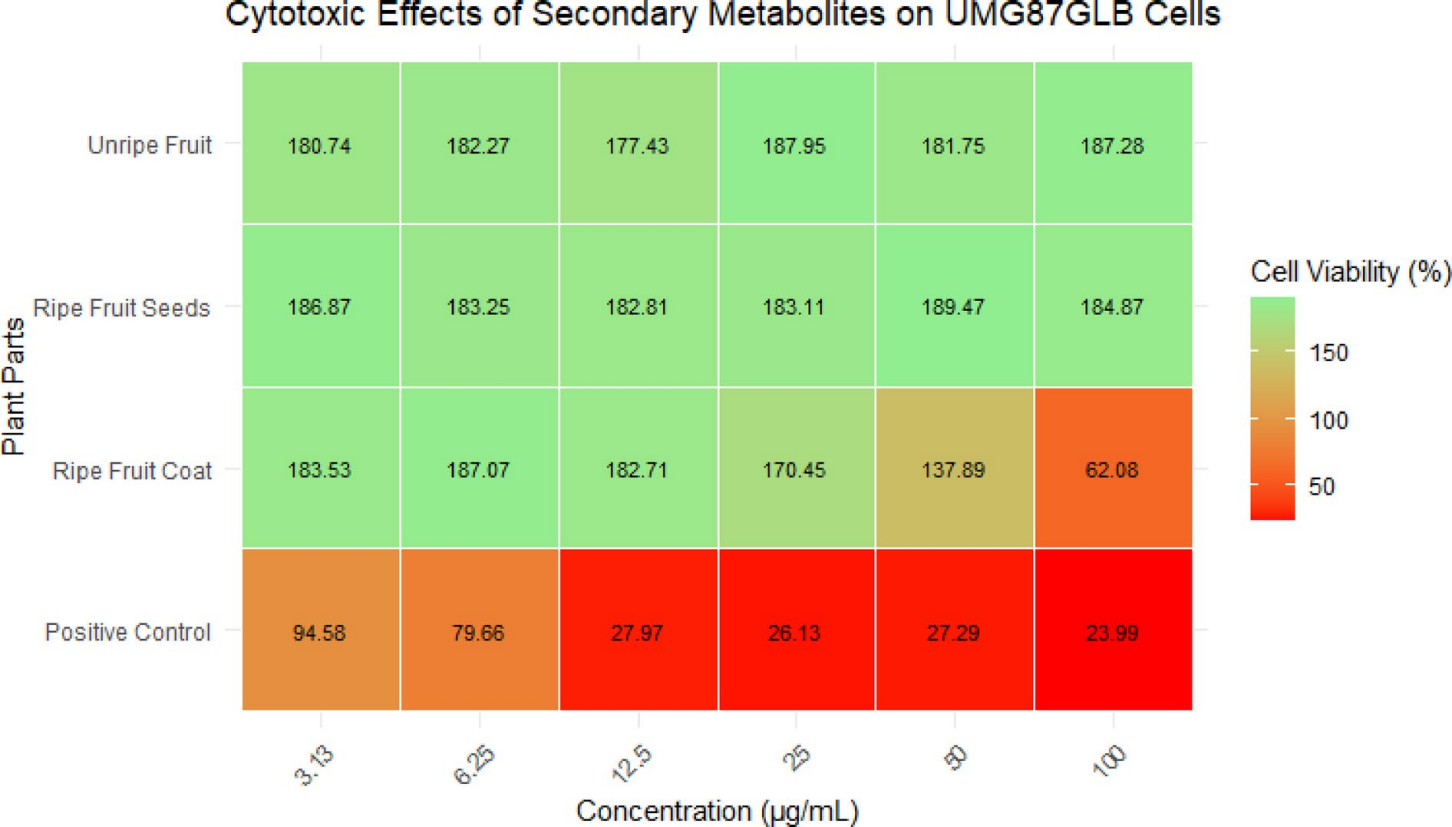
Heatmap of the cytotoxic effects of secondary metabolites from fruits of *S. mauritianum* on U-87 MG glioblastoma cells tested at different concentrations ranging from 100–3.13 µg/mL. Auranofin was used as the positive control.

The Cytotoxic effects of secondary metabolites from fruits of *S. mauritianum* on U-87 MG glioblastoma cells tested at different concentrations are shown in Fig. 4. The results show a range of cell viability percentages across different concentrations. The cytotoxic effect is inversely proportional to the cell viability values; lower percentages indicate higher cytotoxicity. Among the tested plant parts, the crude extract from the ripe fruit coat demonstrated the most significant cytotoxic effect, as reflected by the lowest cell viability values across all tested concentrations. The significant cytotoxic impact of the ripe fruit coat at lower concentrations indicates the presence of bioactive compounds with potential anticancer properties, potentially through apoptosis induction or cell cycle arrest mechanisms. The lack of significant cytotoxicity in other plant parts suggests lower metabolite concentration or the absence of key active compounds. This suggests that the metabolites in the ripe fruit coat are particularly potent in reducing glioblastoma cell viability, potentially indicating a strong anticancer activity. In contrast, other plant parts did not show comparable cytotoxicity, as their cell viability percentages were higher, indicating less effectiveness in inhibiting cell growth. The data highlight that the ripe fruit coat’s crude extract from *S. mauritianum* notably reduces the viability of U-87 MG glioblastoma cells, making it a candidate for further investigation in cancer research for its potential therapeutic applications. The two-way ANOVA results indicate that plant parts and extract concentration significantly (*p* < 0.05) affect cell viability, with their effects being interdependent. Assumptions of normality and homogeneity of variance were met, confirming the reliability of the analysis. Post-hoc Tukey tests revealed that all plant parts significantly differed from the positive control, with the ripe fruit coat showing the most potent effect**—**yielding the lowest cell viability, closely aligning with the positive control and suggesting strong cytotoxic potential (see supplementary file S4 – S6). In contrast, seeds and unripe fruits exhibited the highest cell viability. Cell viability decreased as the extract concentration increased, with significant differences (*p* <*0.05*) observed between most concentrations, particularly at lower doses.

The findings from Fig. 4 revealed that secondary metabolites from the fruit coat of *S. mauritianum* were particularly effective in reducing glioblastoma cell viability, potentially indicating a strong anticancer activity. This finding was corroborated by the report of Dasari et al.^54^, who opined that a secondary metabolite from *S. mauritianum* was effective against apoptosis-resistant glioblastoma cells by inducing non-apoptotic cell death. Similarly, several reports concerning *Solanum* species by Koduru et al.^55^, Son et al.^8^ and Jayakumar and Murugan^10^ revealed that specialized metabolites, particularly solasodine, showed potent anticancer activity by promoting apoptosis and inhibiting cell growth (cell viability). While it has been reported that the compound is a potential substance for cancer therapy^8,10,55^, solasodine has also been isolated from other plants besides *S. mauritianum*. A myriad of reports over the past 2–3 decades about the compound showed that it is widespread among plants in the *Solanum* genus^56–60^. Particularly, solasodine is a steroidal alkaloid which has been consistently isolated and identified across multiple *Solanum* species, including *S. nigrum*, *S. lycopersicum*, *S. incanum* and S. *sisymbriifolium*^56,61–63^. Aside from the aforementioned studies, there are also older reports of the isolation of solasodine from different species of the *Solanum* genus^64–67^ and this further buttress that the potency of the compound, as well as other identified compounds, needs further research attention if we must make headway in the quest for new compounds with antiproliferative or cytotoxic and antimicrobial activities. Solasodine, described as a bioactive steroidal alkaloid, shares a striking structural similarity/resemblance to diosgenin—a well-established precursor in steroidal drug synthesis. This similarity underpins its pharmacological significance, contributing to its diverse therapeutic potential. Both compounds are collectively classified as *Solanum* steroids, reflecting their botanical origin and biochemical relatedness with solasodine being successfully synthesized from diosgenin, and extensive research has documented its potent anticancer and antitumor properties, highlighting its promise in oncological drug development^68–71^. Functionally, solasodine exhibits potent anticancer properties and broad-spectrum cytotoxicity through multiple mechanisms, including apoptosis induction, autophagy modulation, and the inhibition of tumour cell proliferation^61,72,73^. For example, Shen et al.^73^ demonstrated that solasodine inhibits the invasion of human lung cancer cells by downregulating miR-21 and MMPs (matrix metalloproteinases) expression (important molecular factors associated with cancer progression, metastasis, and therapy resistance). Xu et al.^72^ aside from apoptosis induction in ovarian cancer cells, solasodine affects autophagy and attenuates metastasis. Interestingly, this compound (solasodine) was also identified amongst the plethora of secondary metabolites found in the fruit coat of *Solanum mauritianum* in our study.

In the context of glioblastoma cytotoxicity, solasodine has been reported to induce apoptosis primarily through mitochondrial dysfunction and caspase activation, involving the regulation of the *Bcl-2/Bax*/*caspase*−3 pathway. Studies have shown that solasodine can trigger the intrinsic apoptotic pathway by increasing *Bax/Bcl-2* ratios, leading to mitochondrial membrane permeabilization and subsequent cytochrome C release^74,75^. This event ultimately activates caspase-9 and caspase-3, promoting programmed cell death in glioblastoma cells. Fan et al.^61^ also reported that solasodine shows potential antitumor activity by inhibiting pancreatic cancer cell proliferation, promoting apoptosis, and stimulating immunity by inhibiting cell proliferation and programmed cell death. Given these mechanisms, solasodine and related Solanum metabolites hold promise for targeting glioblastoma’s highly resistant tumour microenvironment. Future studies should focus on validating these pathways experimentally, particularly concerning the secondary metabolites identified in this study.

**Fig. 5:**
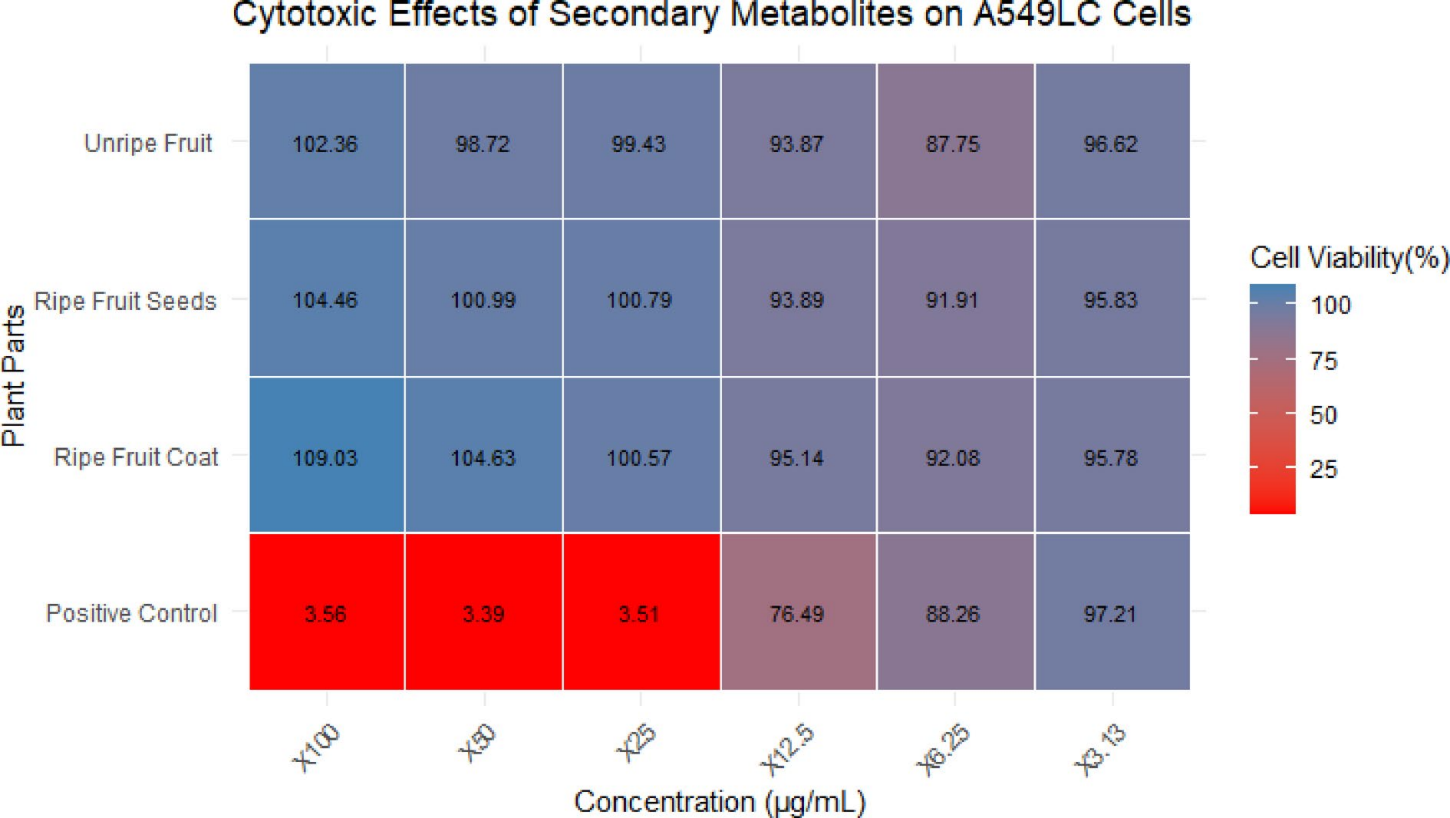
Heatmap of the cytotoxic effects of secondary metabolites from fruits of *S. mauritianum* on A549 lung carcinoma cells tested at different concentrations ranging from 100–3.13 µg/mL. Auranofin was used as the positive control.

The heatmap (Fig. 5) illustrates the effects of secondary metabolites from the fruit of *S. mauritianum* on A549 lung carcinoma cells across a range of concentrations. The values in each cell represent the percentage of cell viability, with higher values indicating lower cytotoxic activity and vice versa. The heatmap shows that none of the secondary metabolites from the different plant parts exhibited significant cytotoxic effects against A549 cells at any tested concentrations. Cell viability remained relatively high across all treatments, with values consistently above 87%, suggesting that the metabolites did not significantly reduce the viability of the A549 lung carcinoma cells. The positive control, which is expected to demonstrate cytotoxicity, showed very low cell viability values (below 4%), confirming the effectiveness of the control treatment.

In contrast, the crude extracts from the unripe fruit, ripe fruit seeds, and ripe fruit coat of *S. mauritianum* maintained cell viability close to or above 95%, indicating that these extracts do not exert a cytotoxic effect on A549 cells under the conditions tested. Overall, the data suggest that the secondary metabolites from the fruit of *S. mauritianum* do not possess cytotoxic properties against A549 lung carcinoma cells at the concentrations tested, highlighting a lack of anticancer activity in this context. More importantly, secondary metabolites from the fruit of *S. mauritianum* possess selective anticancer activity as it was infective against brain cells and not on lung cells.

The result of the cytotoxic activity of crude extract from fruits of *S. mauritianum* against lung carcinoma cells revealed that the secondary metabolites do not possess any cytotoxic properties against A549 lung carcinoma cells at the tested concentrations, thereby highlighting the lack of anticancer activity in this context. In contrast to glioblastoma cells, where it was found that extracts from the fruit coat were found to have cytotoxic activity, it further revealed that crude extracts from fruits of *S. mauritianum* may only be effective against glioblastoma cell lines but not lung carcinoma cell lines thereby shedding light on the specificity, effectiveness and potential for use in clinical trials against cancer. This observation underscores the potential of these extracts in targeting glioblastoma-specific pathways, thereby reducing the likelihood of systemic toxicity and off-target effects, which are common challenges in chemotherapy. Selectivity in cytotoxic activity is desirable in anticancer drug development, as it enhances therapeutic efficacy while minimizing harm to non-cancerous tissues. Moreover, the observed specificity suggests that the active metabolites present in *S. mauritianum* extracts may interact with molecular pathways unique to glioblastoma cells. Future studies should focus on fractionating the crude extract to isolate and characterize the specific bioactive compounds responsible for this selective activity. Such an approach will help elucidate the underlying mechanisms of action and determine whether these metabolites can be developed into targeted glioblastoma therapies. Further investigations into their mode of action, including apoptosis induction and inhibition of glioblastoma-associated signalling pathways, will provide deeper insights into their therapeutic potential. Based on the findings obtained in this study, the results of low cytotoxicity (indicated by high cell viability) against lung carcinoma cells by crude extracts of *S. mauritianum* were consistent with the report of Uche-Okereafor et al.^38^, who opined that crude extracts from the same plant increase the proliferation of lung carcinoma cells. At variance with the result obtained in this study were the reports of Mishra et al.^76^, who opined that seed extracts of *Z. mauritiana* induced apoptosis by inhibiting the proliferation of lung carcinoma cells in a dose-dependent manner. Clearly, the variation in findings is due to the fact that different plants were assayed for their cytotoxic activities. More so, Tapfuma et al.^31^ evaluated the cytotoxicity of crude extracts against lung carcinoma cell lines and observed that there was increased cell proliferation, as seen in this study, which further adds to the hypothesis that crude extract of *S. mauritianum* fruit component exhibits selective cytotoxicity and is not effective against lung carcinoma cell line.

**Fig. 6:**
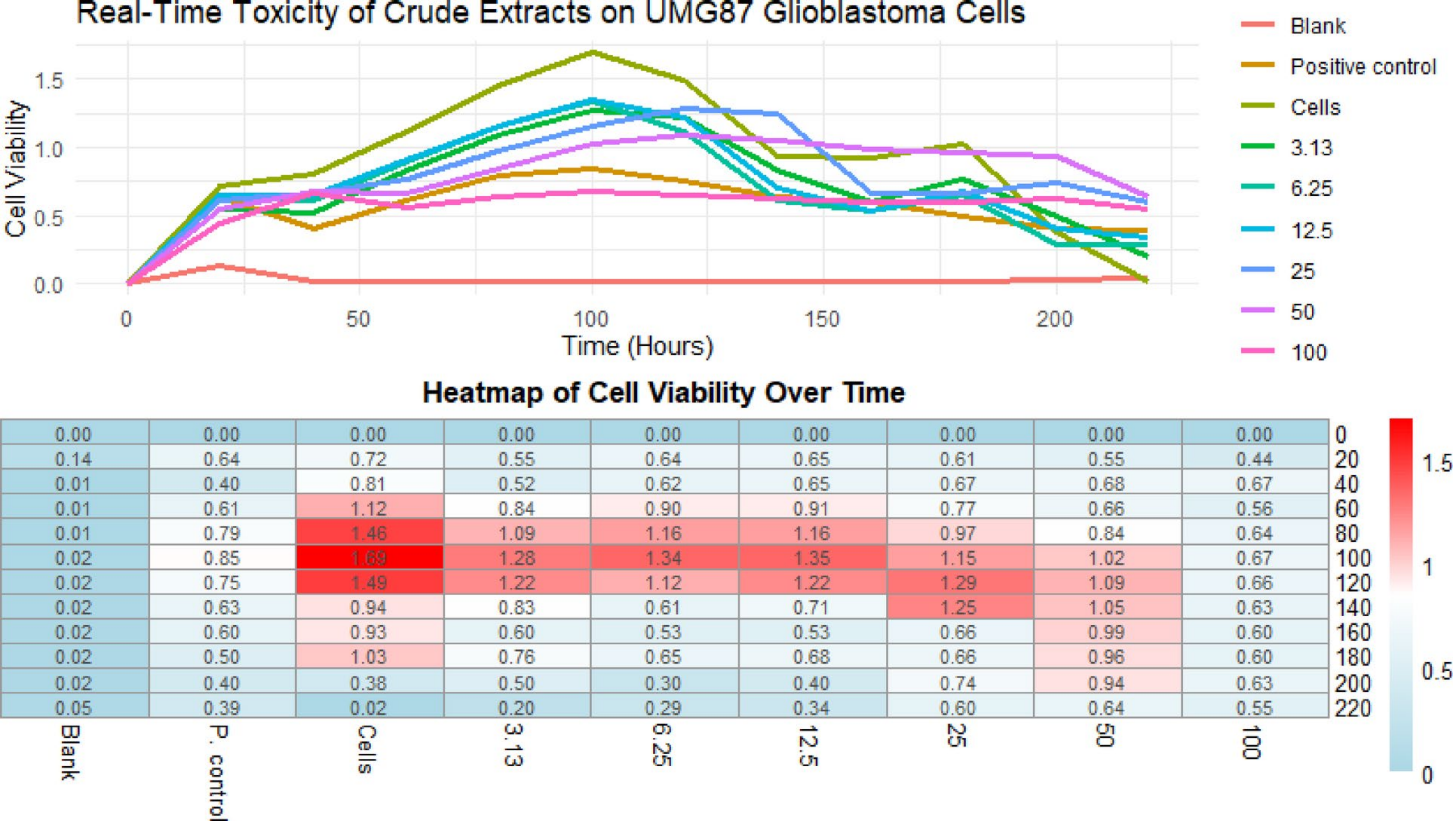
Cowplot of the real-time cytotoxic effects of crude extracts derived from the fruit coat of *S. mauritianum* on U-87 MG glioblastoma cells.

For the *S. mauritianum* fruit component with anticancer activity for its crude extract against glioblastoma cells, the cowplot (Fig. 6) illustrates the real-time cytotoxic effects of crude extracts derived from the fruit coat of *S. mauritianum* on U-87 MG glioblastoma cells. The assay provides a dynamic visualization of the time-dependent cellular response, shedding light on the potential anticancer properties of *S. mauritianum* fruit coat extracts. The result showcases the cellular viability over time, allowing for precise tracking of how the extract influences glioblastoma cell proliferation and cytotoxicity of the extract by extension. A progressive decline in cell viability was observed, indicating the potency of the fruit coat extract in inducing cytotoxic effects on the glioblastoma cells. The real-time monitoring adds a layer of depth, illustrating not only the extent of cytotoxicity but also the kinetics, which are crucial for understanding the extract’s potential for therapeutic application. The key insights from the cowplot (Fig. 6) include the onset of cytotoxicity, its intensity over time, and the relationship between extract concentration and cellular response. Notably, the plot delineates a clear dose-dependent inhibition of U-87 MG glioblastoma cells, with higher extract concentrations accelerating cell death. This suggests that the crude fruit coat extract targets essential cellular pathways, potentially disrupting cell division and survival mechanisms in a manner akin to conventional anticancer agents.

As earlier stated, the findings in the study correspond to the aforementioned reports, where the activity of *S. mauritianum* fruit was found to have potential anticancer properties^10,41,45,55,56,58,76^. The findings effectively demonstrate the cytotoxic capabilities of *S. mauritianum* fruit coat extracts, positioning it as a promising candidate for further investigation in glioblastoma therapy. The real-time data emphasizes the extract’s potency and provides valuable insights into the temporal dynamics of its anticancer effects, which could guide future in vivo studies and potential clinical applications.

### Limitations of the study

While our study provides valuable insights into the secondary metabolite profile of *S. mauritianum*, we acknowledge several limitations, which include the extraction solvent (mildly), failure to analyze green fruit coat and variability in extraction efficiency as aforementioned. Though effective in extracting antibacterial compounds, the 50:50 methanol and chloroform solvent combination inherently favours mid-polar and nonpolar metabolites. This may have resulted in excluding highly polar compounds, potentially limiting the diversity of metabolites identified. Variability in extraction efficiency due to differences in solvent polarity, sample preparation techniques, and metabolite stability could also influence the reproducibility of results. Additionally, this study did not analyze the green fruit coat, which may have led to the omission of unique bioactive compounds in unripe tissues. Since different metabolic pathways are active during fruit maturation, the absence of data from green fruit coats limits the comprehensiveness of the metabolite profiling. Furthermore, the antibacterial bioassays’ specificity may have influenced the observed activity, as certain metabolites might exhibit selective effects against specific bacterial strains. Future studies should explore a broader range of solvent combinations to ensure a more comprehensive metabolite profile and expand bioassay screenings to include additional bacterial and fungal pathogens to fully evaluate the pharmacological potential of *S. mauritianum* metabolites.

## Conclusion

The present study thoroughly examines the secondary metabolites in different parts of *Solanum mauritianum* fruits, shedding light on their distribution and potential therapeutic applications. The metabolite profiles showed that the ripe fruit coat contained the most unique metabolites, with 15 distinct compounds. This plant part demonstrated a rich diversity of bioactive metabolites, likely contributing to its higher bioactive potential. The ripe and green fruit seeds followed in terms of metabolite richness, but to a lesser extent, with the latter containing only five unique metabolites. The overlap of metabolites across different plant parts—specifically the eight common compounds—indicates that these core metabolites may play essential roles in the plant’s overall biochemical processes, possibly influencing its adaptation and defence mechanisms. The classification of these metabolites highlighted the prominence of alkaloids and terpenoids, constituting more than half of the identified metabolites. The cytotoxicity results against U-87 MG glioblastoma cells were particularly noteworthy. The ripe fruit coat extract exhibited a significant reduction in cell viability, especially at higher concentrations, indicating a potent anticancer activity. This is of great importance, considering the aggressive nature of glioblastoma and the need for more effective treatments. The real-time cytotoxicity analysis further supported this finding, showing an apparent dose-dependent effect of the ripe fruit coat extract on U-87 MG cells.

In contrast, the lack of significant cytotoxicity against A549 lung carcinoma cells was unexpected, especially given the strong activity against glioblastoma cells. This selective anticancer effect suggests that the secondary metabolites of *S. mauritianum* may have targeted mechanisms that specifically affect brain tumour cells, potentially offering a more tailored therapeutic approach. The high cell viability in A549 cells across all concentrations tested indicates that the extracts may not have broad-spectrum anticancer activity but rather a selective effect, which could be advantageous in reducing side effects in clinical applications.

The selective activity against glioblastoma and the real-time cytotoxicity data pave the way for future investigations into the mechanisms underlying this effect. Moreover, the rich diversity of secondary metabolites in the fruit coat suggests that *S. mauritianum* could be a valuable resource for discovering new bioactive compounds. Future studies should focus on isolating and characterizing these metabolites, exploring their mechanisms of action, and conducting in vivo studies to validate their therapeutic potential in cancer treatment.

## Electronic supplementary material

Below is the link to the electronic supplementary material.

Supplementary Material 1

## Data Availability

The datasets used and/or analyzed during the current study are available from the corresponding author upon reasonable request.
