# Supplementary material for: Antibacterial and anticancer properties of Solanum mauritianum fruit components analyzed using LC-QTOF-MS/MS
Source: Sci Rep. 2025 May 14;15:16698. doi: 10.1038/s41598-025-01348-w (PMC12078596; doi:10.1038/s41598-025-01348-w)
Supplement: Supplementary file 1 — Supplementary Material 1 [file 41598_2025_1348_MOESM1_ESM.docx]

Supplementary Material

Figure S1. Extracted ion chromatogram (EIC) of secondary metabolites from *Solanum mauritianum* ripe fruits coat – S1 RFC (left) and their MS^2^ fragments (right).

| **EIC: y axis = Intensity; x axis = Time (min)** | **MS^2^: y axis = Intensity; x axis = *m/z* (Mass to charge ratio)** |
| --- | --- |
| Base Peak Chromatogram  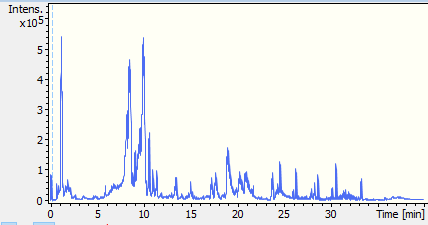 | 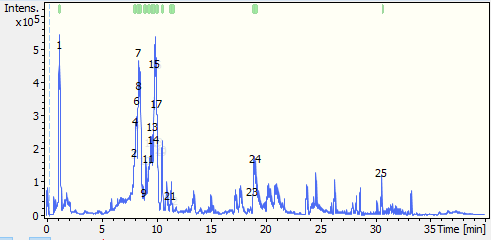 |
| Cardiospermin  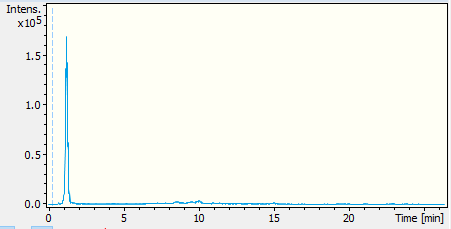  **EIC (*m/z* 276.0970; +MS)** | 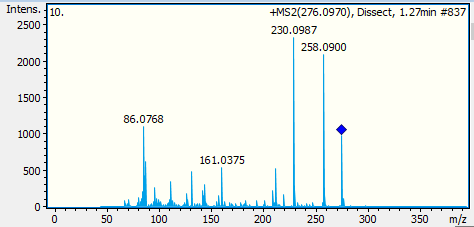  **Fragments *m/z* 276.0970; +MS/MS** |
| Furofoline I  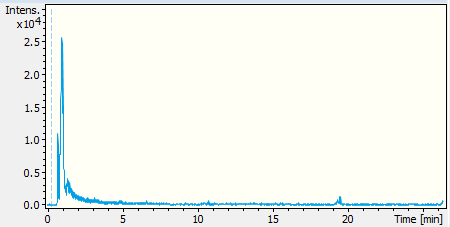  **EIC (*m/z* 266.0796; +MS)** | 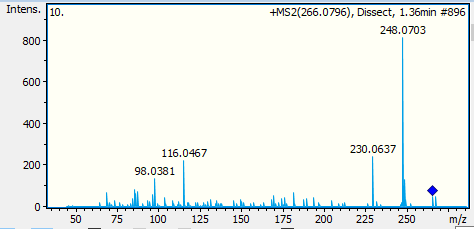  **Fragments *m/z* 266.0796; +MS/MS** |
| Riccionidin A  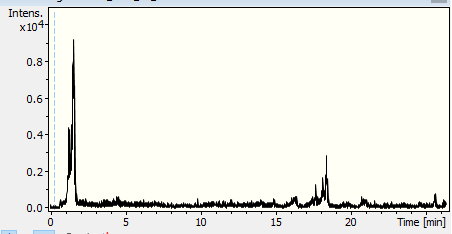  **EIC (*m/z* 286.0453; +MS)** | 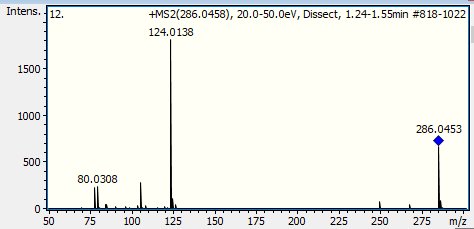  **Fragments *m/z* 286.0453; +MS/MS** |
| Bergenin  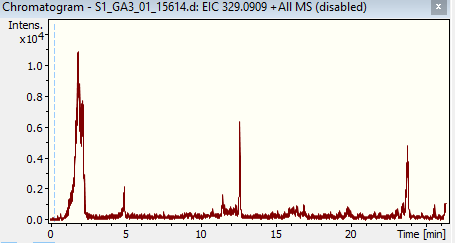  **EIC (*m/z* 329.0909; +MS)** | 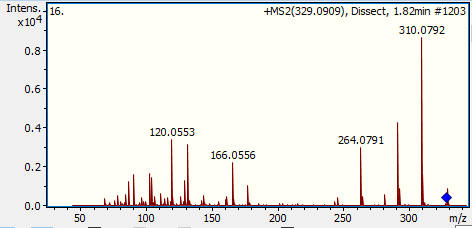  **Fragments *m/z* 329.0909; +MS/MS** |
| Cusparine  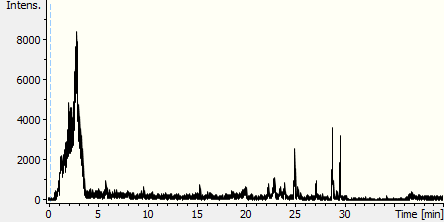  **EIC (*m/z* 308.1209; +MS)** | 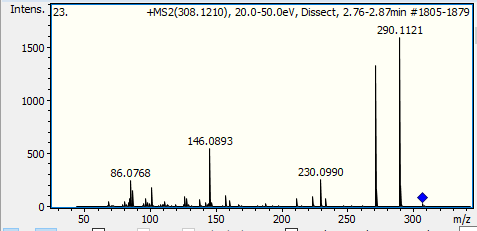  **Fragments *m/z* 308.1209; +MS/MS** |
| Theogallin  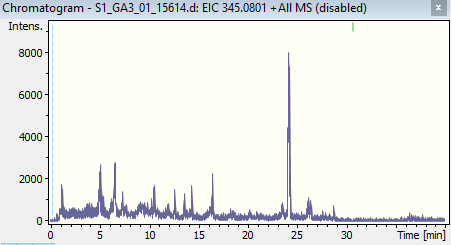  **EIC (*m/z* 345.0801; +MS)** | 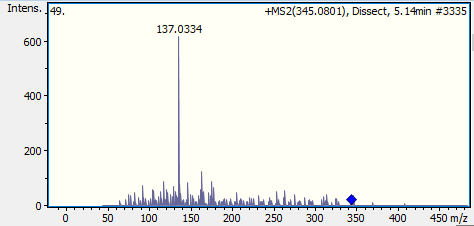  **Fragments *m/z* 345.0801; +MS/MS** |
| Homoeriodictyol chalcone  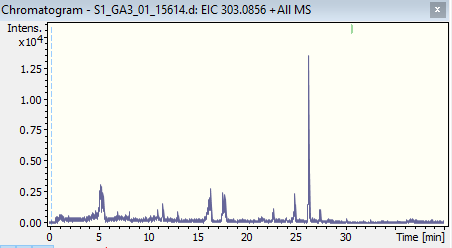  **EIC (*m/z* 303.0856; +MS)** | 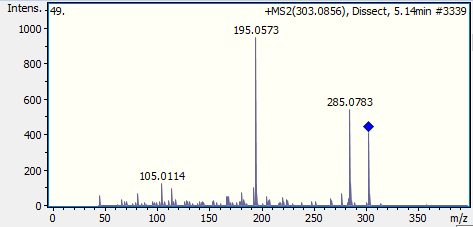  **Fragments *m/z* 303.0856; +MS/MS** |
| Berberastine  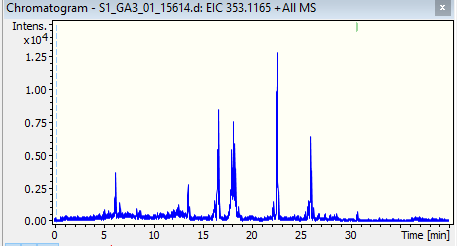  **EIC (*m/z* 353.1165; +MS)** | 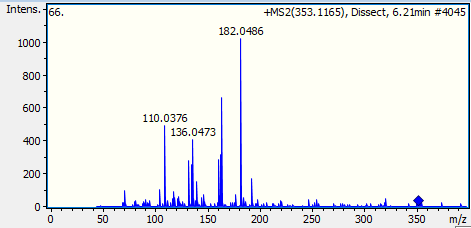  **Fragments *m/z* 353.1165; +MS/MS** |
| Scopoletin  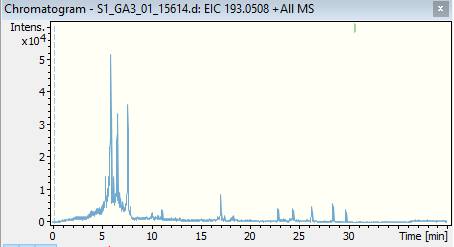  **EIC (*m/z* 193.0508; +MS)** | 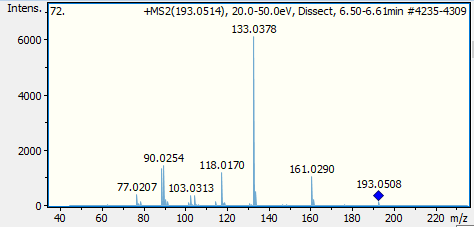  **Fragments *m/z* 193.0508; +MS/MS** |
| Lysergic acid  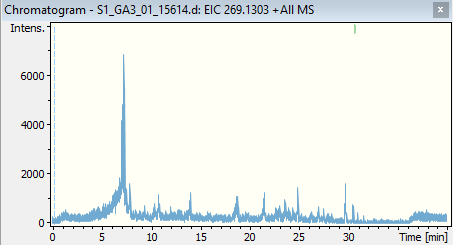  **EIC (*m/z* 269.1303; +MS)** | 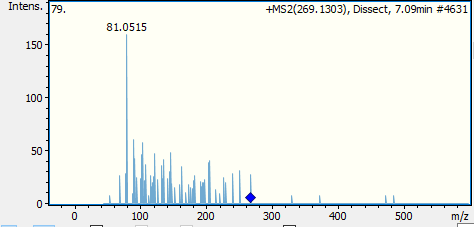  **Fragments *m/z* 269.1303; +MS/MS** |
| Glycophymoline  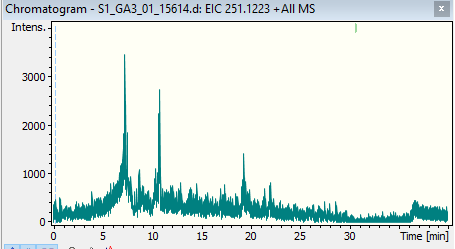  **EIC (*m/z* 251.1223; +MS)** | 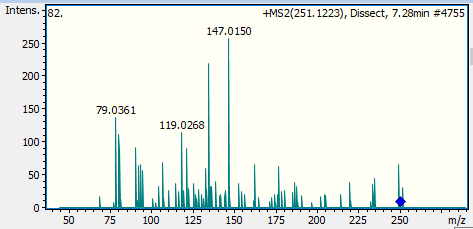  **Fragments *m/z* 251.1223; +MS/MS** |
| Solasonine  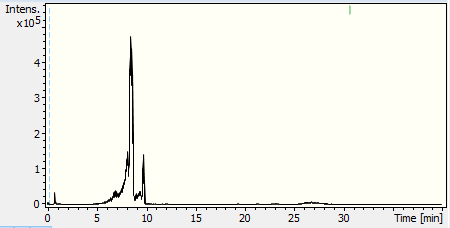  **EIC (*m/z* 884.3903; +MS)** | 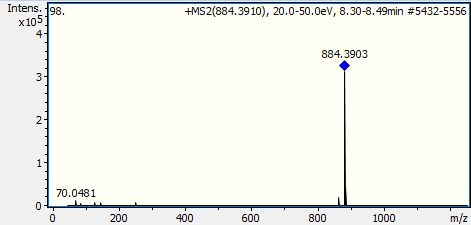  **Fragments *m/z* 884.3903; +MS/MS** |
| α-Solanine  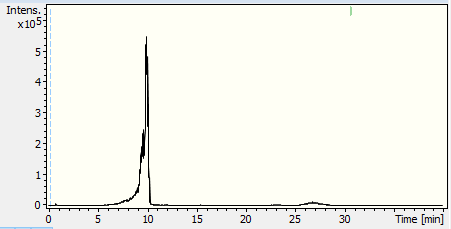  **EIC (*m/z* 868.3953; +MS)** | 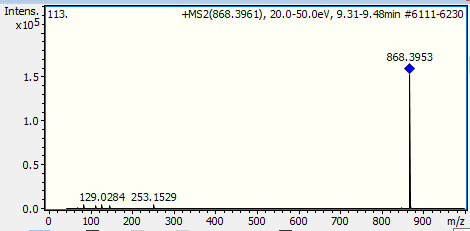  **Fragments *m/z* 868.3953; +MS/MS** |
| Imperialine  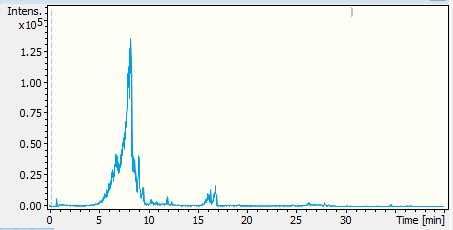  **EIC (*m/z* 430.2686; +MS)** | 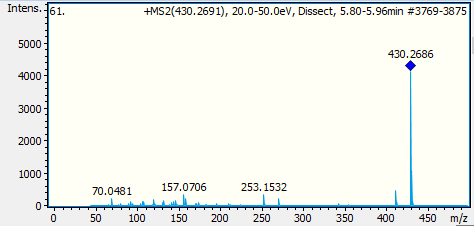  **Fragments *m/z* 430.2686; +MS/MS** |
| Genipin  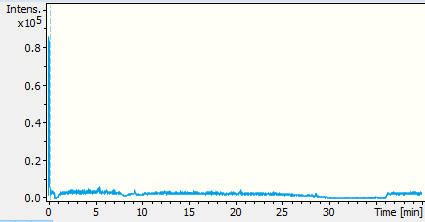  **EIC (*m/z* 227.1021; +MS)** | 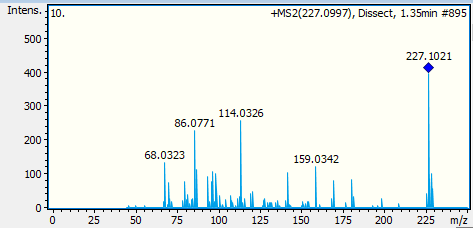  **Fragments *m/z* 227.1021; +MS/MS** |
| Solasodine  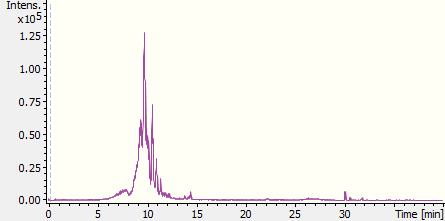  **EIC (*m/z* 414.2751; +MS)** | 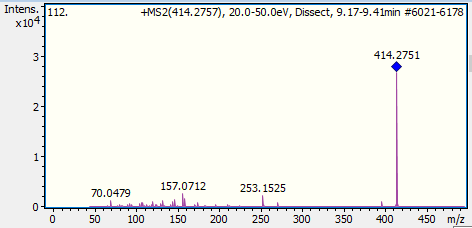  **Fragments *m/z* 414.2751; +MS/MS** |
| α-Ergocryptine  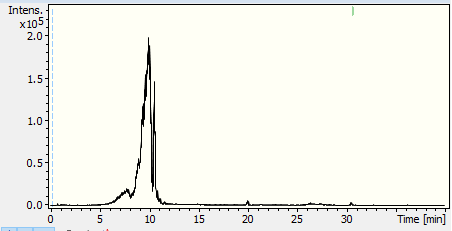  **EIC (*m/z* 576.3100; +MS)** | 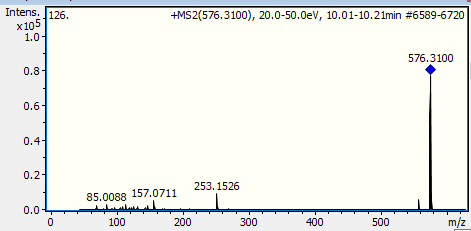  **Fragments *m/z* 576.3100; +MS/MS** |
| Callicarpone  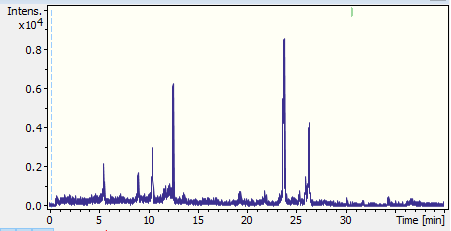  **EIC (*m/z* 333.2115; +MS)** | 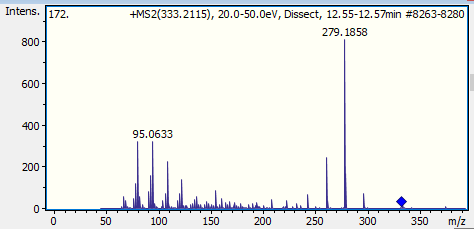  **Fragments *m/z* 333.2115; +MS/MS** |
| 10-Deoxysarpagine  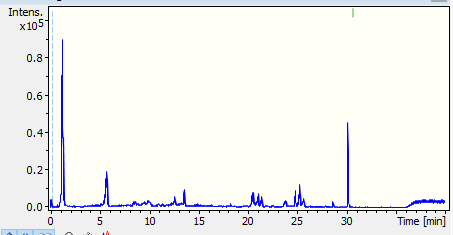  **EIC (*m/z* 295.1791; +MS)** | 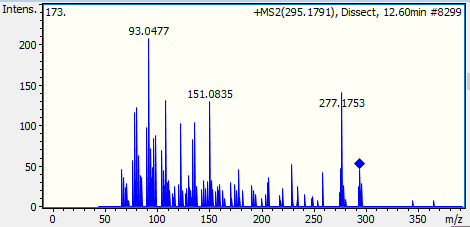  **Fragments *m/z* 295.1791; +MS/MS** |
| Montanol  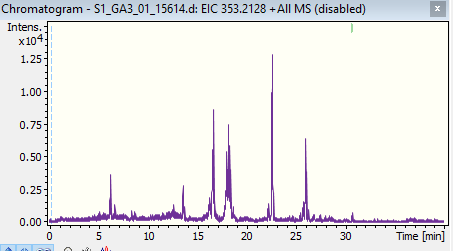  **EIC (*m/z* 353.2128; +MS)** | 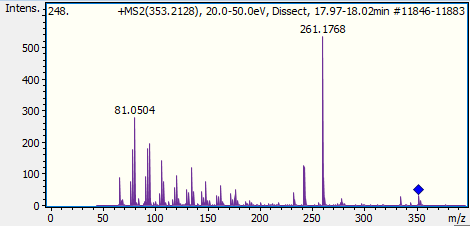  **Fragments *m/z* 353.2128; +MS/MS** |
| Hypercalin B  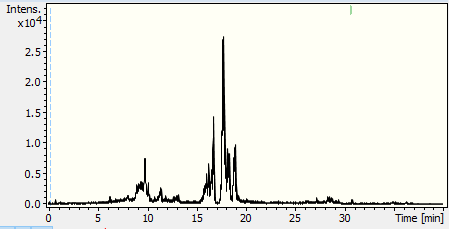  **EIC (*m/z* 519.3100; +MS)** | 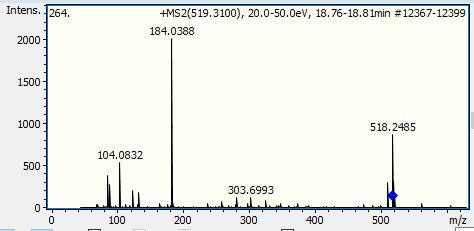  **Fragments *m/z* 519.3100; +MS/MS** |
| Eugenin  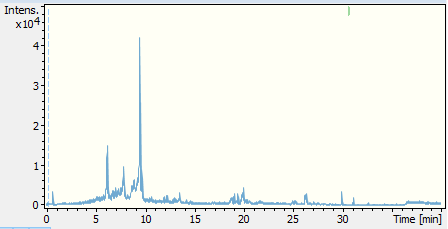  **EIC (*m/z* 207.0642; +MS)** | 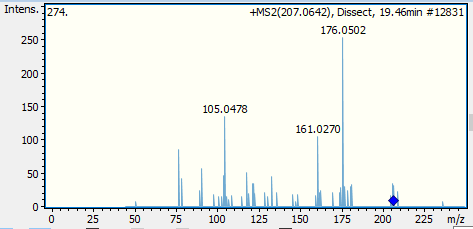  **Fragments *m/z* 207.0642; +MS/MS** |
| Thalicarpine  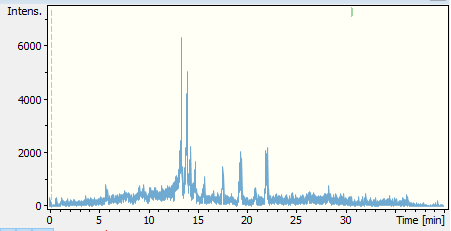  **EIC (*m/z* 697.3305; +MS)** | 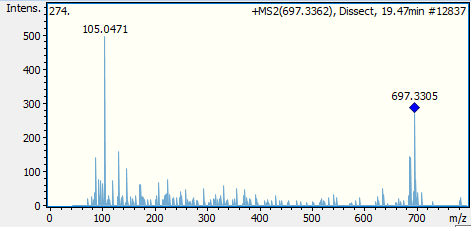  **Fragments *m/z* 697.3305; +MS/MS** |
| Anatabine  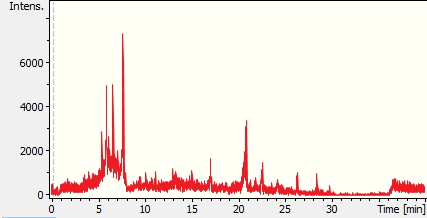  **EIC (*m/z* 161.0641; +MS)** | 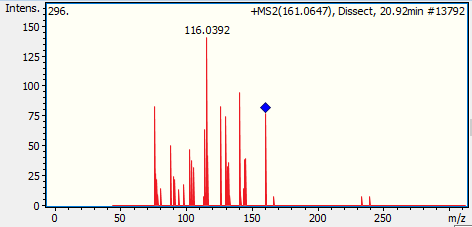  **Fragments *m/z* 161.0641; +MS/MS** |
| Ibogamine  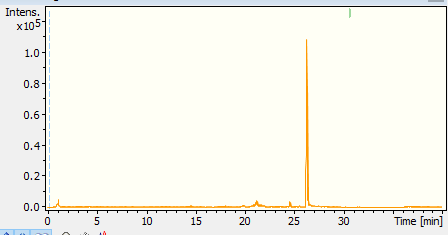  **EIC (*m/z* 281.1998; +MS)** | 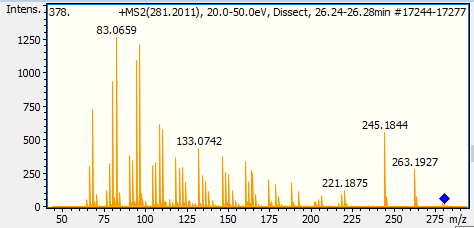  **Fragments *m/z* 281.1998; +MS/MS** |
| Ibogaine  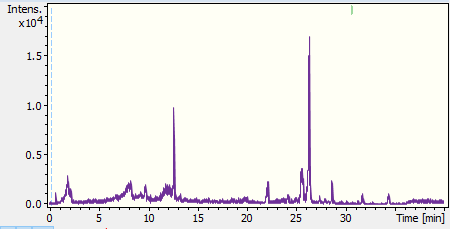  **EIC (*m/z* 311.2077; +MS)** | 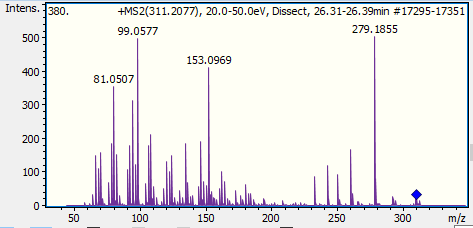  **Fragments *m/z* 311.2077; +MS/MS** |
| Loganin  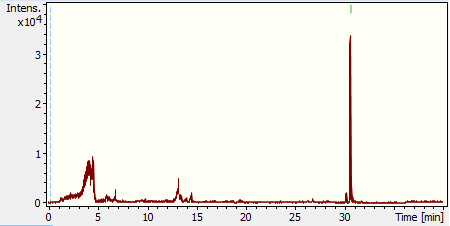  **EIC (*m/z* 391.1497; +MS)** | 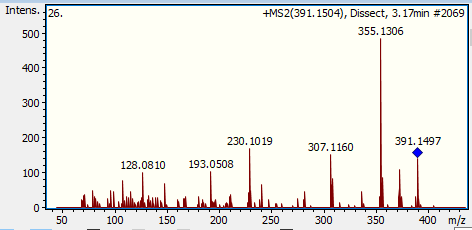  **Fragments *m/z* 391.1497; +MS/MS** |
| Tingenone  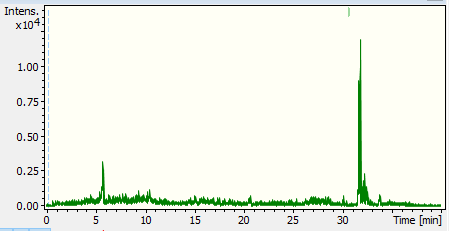  **EIC (*m/z* 421.2636; +MS)** | 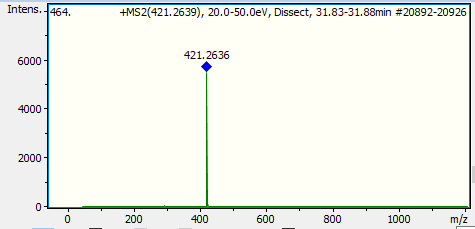  **Fragments *m/z* 421.2636; +MS/MS** |

Figure S2. Extracted ion chromatogram (EIC) of secondary metabolites from *Solanum mauritianum* ripe fruit seeds – S2 RFS (left) and their MS^2^ fragments (right).

| **EIC: y axis = Intensity; x axis = Time (min)** | **MS^2^: y axis = Intensity; x axis = *m/z* (Mass to charge ratio)** |
| --- | --- |
| Base Peak Chromatogram (BPC)  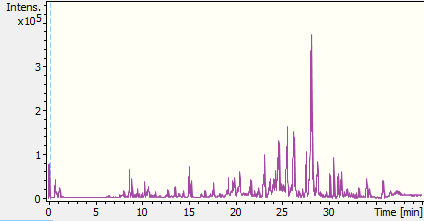 | 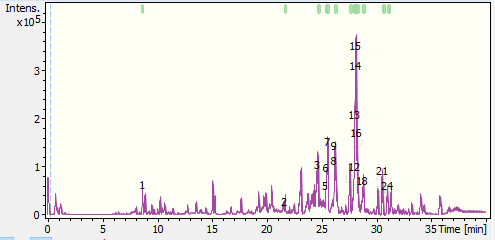 |
| Scopoletin  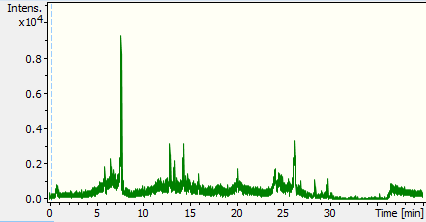  **EIC (*m/z* 193.0514; +MS)** | 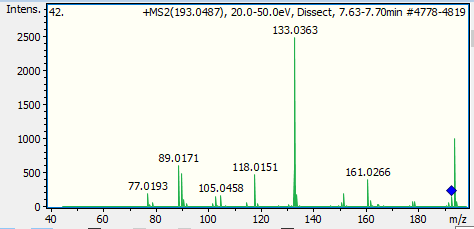  **Fragments *m/z* 193.0514; +MS/MS** |
| Danielone  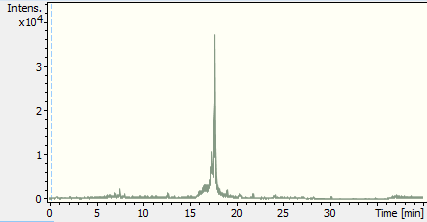  **EIC (*m/z* 213.0723; +MS)** | 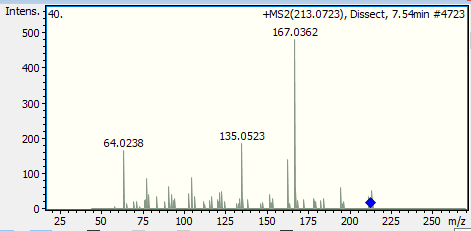  **Fragments *m/z* 213.0723; +MS/MS** |
| Solasonine  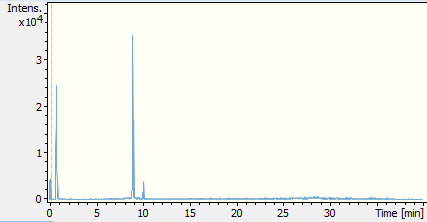  **EIC (*m/z* 884.3784; +MS)** | 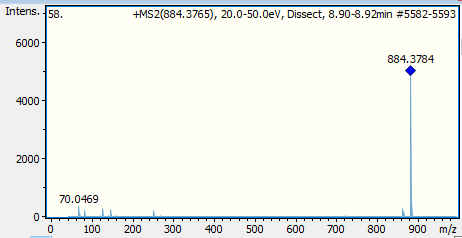  **Fragments *m/z* 884.3784; +MS/MS** |
| α-Solanine  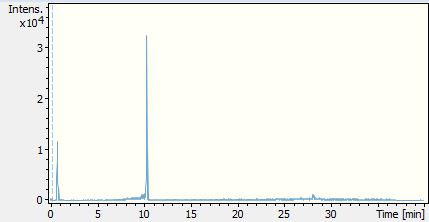  **EIC (*m/z* 868.3878; +MS)** | 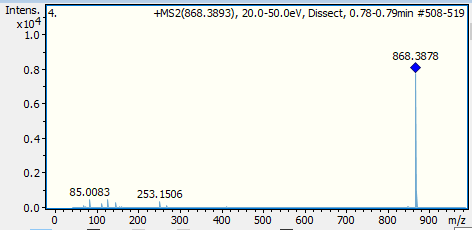  **Fragments *m/z* 868.3878; +MS/MS** |
| Imperialine  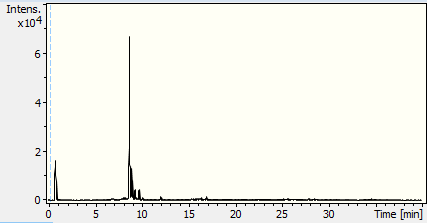  **EIC (*m/z* 430.2637; +MS)** | 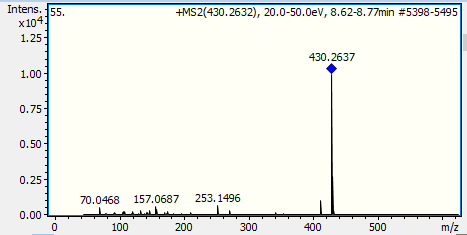  **Fragments *m/z* 430.2637; +MS/MS** |
| Genipin  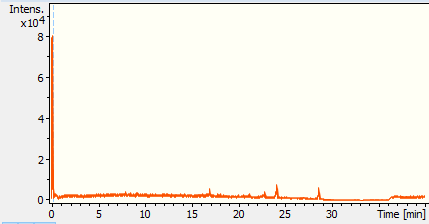  **EIC (*m/z* 227.0971; +MS)** | 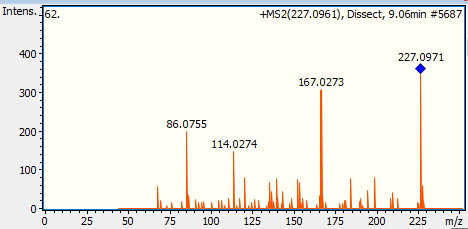  **Fragments *m/z* 227.0971; +MS/MS** |
| α-Ergocryptine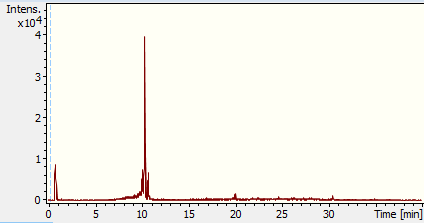  **EIC (*m/z* 576.3026; +MS)** | 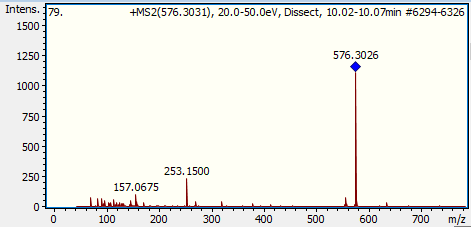  **Fragments *m/z* 576.3026; +MS/MS** |
| Solasodine  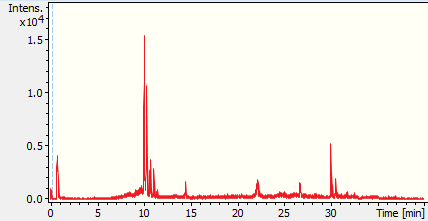  **EIC (*m/z* 414.2708; +MS)** | 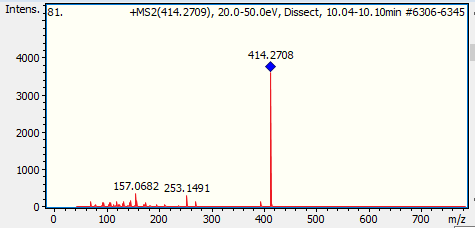  **Fragments *m/z* 414.2708; +MS/MS** |
| Podolide  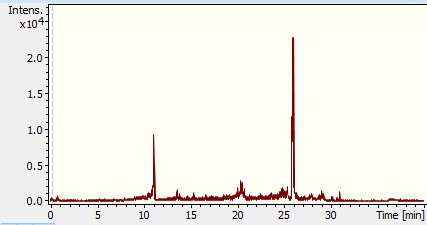  **EIC (*m/z* 331.1619; +MS)** | 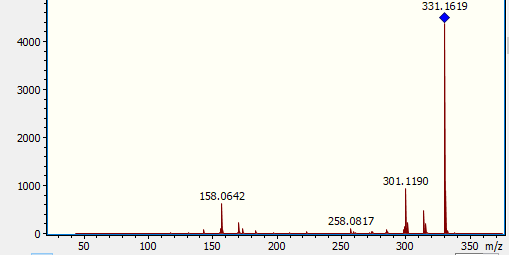  **Fragments *m/z* 331.1619; +MS/MS** |
| Esculetin  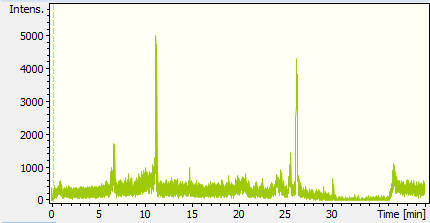  **EIC (*m/z* 179.0357; +MS)** | 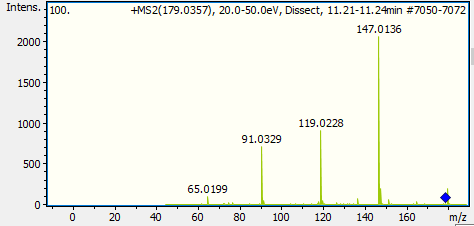  **Fragments *m/z* 179.0357; +MS/MS** |
| 10-Deoxysarpagine  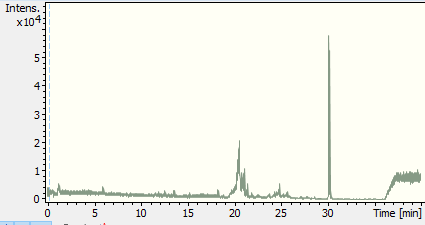  **EIC (*m/z* 295.1791; +MS)** | 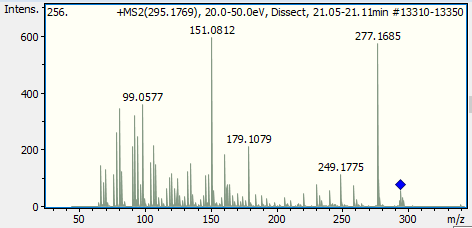  **Fragments *m/z* 295.1791; +MS/MS** |
| Glycobismine A  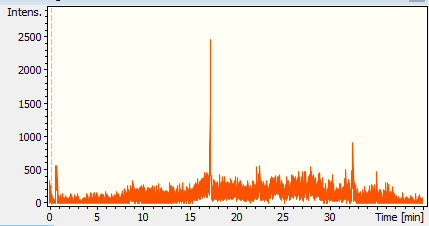  **EIC (*m/z* 603.2512; +MS)** | 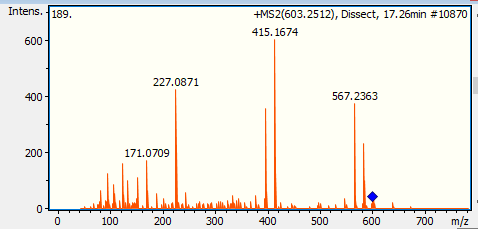  **Fragments *m/z* 603.2512; +MS/MS** |
| Montanol  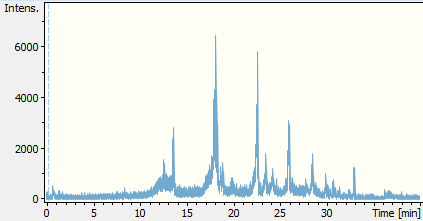  **EIC (*m/z* 353.2128; +MS)** | 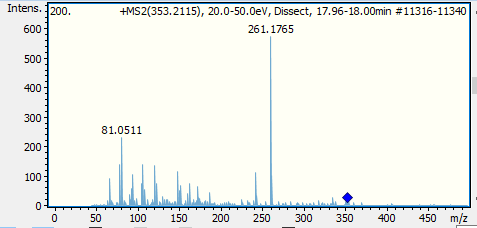  **Fragments *m/z* 353.2128; +MS/MS** |
| Coniferyl alcohol  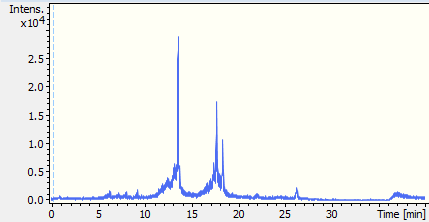  **EIC (*m/z* 181.0874; +MS)** | 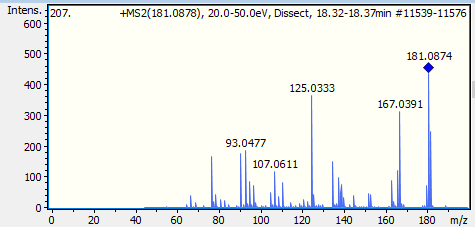  **Fragments *m/z* 181.0874; +MS/MS** |
| Ribalinium  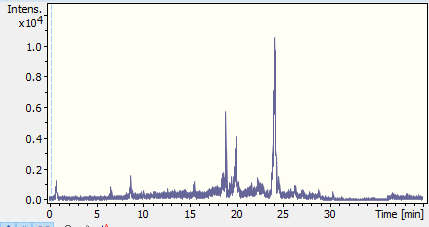  **EIC (*m/z* 291.1457; +MS)** | 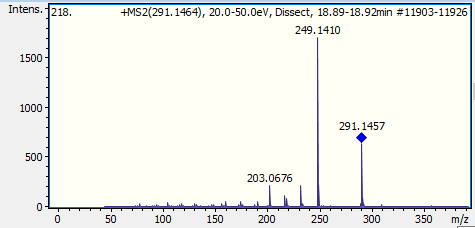  **Fragments *m/z* 291.1457; +MS/MS** |
| Rhododendrin  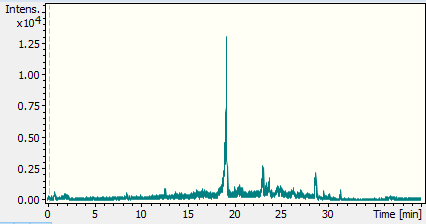  **EIC (*m/z* 329.1607; +MS)** | 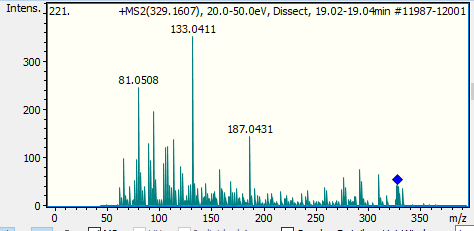  **Fragments *m/z* 329.1607; +MS/MS** |
| Eugenin  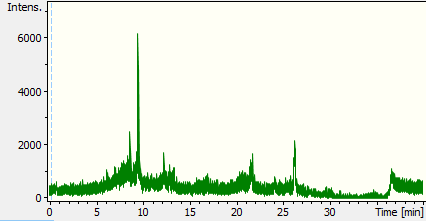  **EIC (*m/z* 207.0642; +MS)** | 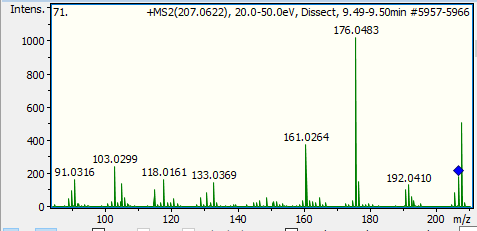  **Fragments *m/z* 207.0642; +MS/MS** |
| Hetisine  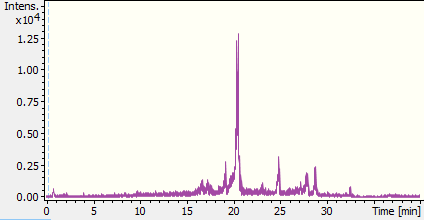  **EIC (*m/z* 330.2096; +MS)** | 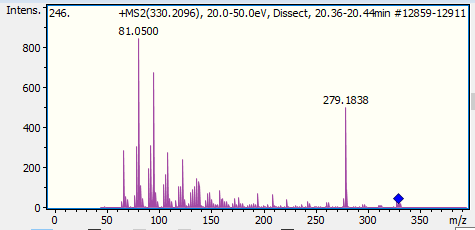  **Fragments *m/z* 330.2096; +MS/MS** |
| Anatabine  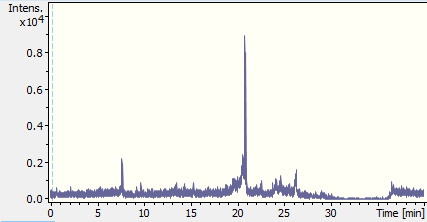  **EIC (*m/z* 161.0639; +MS)** | 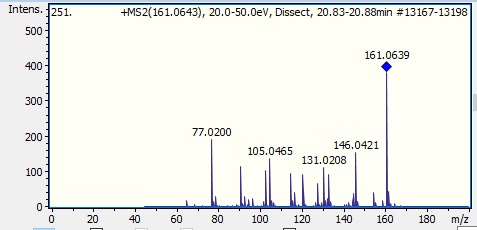  **Fragments *m/z* 161.0639; +MS/MS** |
| Tuliposide A  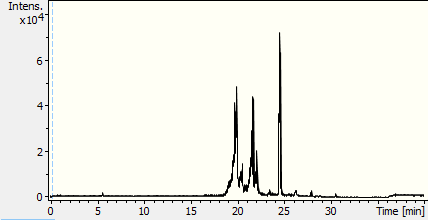  **EIC (*m/z* 276.0970; +MS)** | 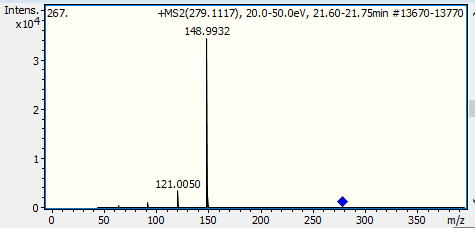  **Fragments *m/z* 276.0970; +MS/MS** |
| Vasconine    **EIC (*m/z* 267.1281; +MS)** | **Fragments *m/z* 267.1281; +MS/MS** |
| Columbin    **EIC (*m/z* 359.1517; +MS)** | **Fragments *m/z* 359.1517; +MS/MS** |
| Lycorine    **EIC (*m/z* 288.1257; +MS)** | **Fragments *m/z* 288.1257; +MS/MS** |
| Cannabielsoin    **EIC (*m/z* 331.2300; +MS)** | **Fragments *m/z* 331.2300; +MS/MS** |
| Absinthin    **EIC (*m/z* 497.2856; +MS)** | **Fragments *m/z* 497.2856; +MS/MS** |
| Clivoline    **EIC (*m/z* 406.1889; +MS)** | **Fragments *m/z* 406.1889; +MS/MS** |

Figure S3. Extracted ion chromatogram (EIC) of secondary metabolites from *Solanum mauritianum* unripe (green) fruit seeds – S3 GFS (left) and their MS^2^ fragments (right).

| **EIC: y axis = Intensity; x axis = Time (min)** | **MS^2^: y axis = Intensity; x axis = *m/z* (Mass to charge ratio)** |
| --- | --- |
| Base Peak Chromatogram (BPC) |  |
| Loganin    **EIC (*m/z* 391.1504; +MS)** | **Fragments *m/z* 391.1504; +MS/MS** |
| Cimifugin    **EIC (*m/z* 307.1253; +MS)** | **Fragments *m/z* 307.1253; +MS/MS** |
| Scopoletin    **EIC (*m/z* 193.0514; +MS)** | **Fragments *m/z* 193.0514; +MS/MS** |
| Solasonine | **Fragments *m/z* 884.3840; +MS/MS** |
| α-Solanine    **EIC (*m/z* 868.3905; +MS)** | **EIC (*m/z* 884.3840; +MS)**    **Fragments *m/z* 868.3905; +MS/MS** |
| Imperialine    **EIC (*m/z* 430.2643; +MS)** | **Fragments *m/z* 430.2643; +MS/MS** |
| α-Ergocryptine    **EIC (*m/z* 576.3052; +MS)** | **Fragments *m/z* 576.3052; +MS/MS** |
| Dioscin    **EIC (*m/z* 869.3927; +MS)** | **Fragments *m/z* 869.3927; +MS/MS** |
| Solasodine    **EIC (*m/z* 414.2716; +MS)** | **Fragments *m/z* 414.2716; +MS/MS** |
| 10-Deoxysarpagine    **EIC (*m/z* 295.1791; +MS)** | **Fragments *m/z* 295.1791; +MS/MS** |
| Montanol    **EIC (*m/z* 353.2093; +MS)** | **Fragments *m/z* 353.2093; +MS/MS** |
| Tuliposide A    **EIC (*m/z* 279.1117; +MS)** | **Fragments *m/z* 279.1117; +MS/MS** |
| Lycocernuine    **EIC (*m/z* 279.1843; +MS)** | **Fragments *m/z* 279.1843; +MS/MS** |
| Cannabielsoin    **EIC (*m/z* 331.2300; +MS)** | **Fragments *m/z* 331.2300; +MS/MS** |
| Ibogamine    **EIC (*m/z* 281.1998; +MS)** | **Fragments *m/z* 281.1998; +MS/MS** |
| Cucurbitacin E    **EIC (*m/z* 557.3268; +MS)** | **Fragments *m/z* 557.3268; +MS/MS** |
| Absinthin    **EIC (*m/z* 497.2867; +MS)** | **Fragments *m/z* 497.2867; +MS/MS** |
| Neoquassin    **EIC (*m/z* 391.2211; +MS)** | **Fragments *m/z* 391.2211; +MS/MS** |

**Figure S4. Inhibitory effect of plant extract on UMG 87 glioblastoma cells**

**Figure S5. Dose-response curve of plant extract on UMG 87 glioblastoma cells**

**Figure S6. Effectiveness of plant extract relative to positive control on UMG 87 glioblastoma cells**

summary(anova_model)

Df Sum Sq Mean Sq F value Pr(>F)

Plant_Parts 3 228069 76023 61449 <2e-16 ***

Concentration 5 17386 3477 2811 <2e-16 ***

Plant_Parts:Concentration 15 33976 2265 1831 <2e-16 ***

Residuals 48 59 1

---

Signif. codes: 0 ‘***’ 0.001 ‘**’ 0.01 ‘*’ 0.05 ‘.’ 0.1 ‘ ’ 1

> # Check assumptions

> shapiro.test(residuals(anova_model))

Shapiro-Wilk normality test

data: residuals(anova_model)

W = 0.97729, p-value = 0.2177

> leveneTest(Cell_Viability ~ Plant_Parts * Concentration, data = data_long)

Levene's Test for Homogeneity of Variance (center = median)

Df F value Pr(>F)

group 23 0.2837 0.9991

48

> # Post hoc tests

> TukeyHSD(anova_model, "Plant_Parts")

Tukey multiple comparisons of means

95% family-wise confidence level

Fit: aov(formula = Cell_Viability ~ Plant_Parts * Concentration, data = data_long)

$Plant_Parts

diff lwr upr

Ripe Fruit Coat-Positive Control 107.105278 106.118548 108.0920078

Ripe Fruit Seeds-Positive Control 137.766187 136.779457 138.7529164

Unripe Fruit-Positive Control 135.867782 134.881052 136.8545113

Ripe Fruit Seeds-Ripe Fruit Coat 30.660909 29.674179 31.6476384

Unripe Fruit-Ripe Fruit Coat 28.762503 27.775774 29.7492332

Unripe Fruit-Ripe Fruit Seeds -1.898405 -2.885135 -0.9116754

p adj

Ripe Fruit Coat-Positive Control 0.0e+00

Ripe Fruit Seeds-Positive Control 0.0e+00

Unripe Fruit-Positive Control 0.0e+00

Ripe Fruit Seeds-Ripe Fruit Coat 0.0e+00

Unripe Fruit-Ripe Fruit Coat 0.0e+00

Unripe Fruit-Ripe Fruit Seeds 3.1e-05

> TukeyHSD(anova_model, "Concentration")

Tukey multiple comparisons of means

95% family-wise confidence level

Fit: aov(formula = Cell_Viability ~ Plant_Parts * Concentration, data = data_long)

$Concentration

diff lwr upr p adj

50-100 19.5164319 18.1687521 20.864112 0.0000000

25-100 27.3799981 26.0323183 28.727678 0.0000000

12.5-100 27.7462614 26.3985816 29.093941 0.0000000

6.25-100 43.2788150 41.9311353 44.626495 0.0000000

3.13-100 46.9893614 45.6416816 48.337041 0.0000000

25-50 7.8635663 6.5158865 9.211246 0.0000000

12.5-50 8.2298295 6.8821498 9.577509 0.0000000

6.25-50 23.7623832 22.4147034 25.110063 0.0000000

3.13-50 27.4729295 26.1252497 28.820609 0.0000000

12.5-25 0.3662633 -0.9814165 1.713943 0.9649393

6.25-25 15.8988169 14.5511371 17.246497 0.0000000

3.13-25 19.6093632 18.2616835 20.957043 0.0000000

6.25-12.5 15.5325536 14.1848739 16.880233 0.0000000

3.13-12.5 19.2431000 17.8954202 20.590780 0.0000000

3.13-6.25 3.7105463 2.3628665 5.058226 0.0000000
